# Greener Cleavage of Protected Peptide Fragments from Sieber Amide Resin

**DOI:** 10.1002/open.202200236

**Published:** 2022-12-23

**Authors:** Othman Al Musaimi, Varshitha Gavva, Daryl R. Williams

**Affiliations:** ^1^ Department of Chemical Engineering Imperial College London London SW7 2AZ UK

**Keywords:** fragments condensation, green chemistry, peptide cleavage, protected peptide amide, Sieber amide resin

## Abstract

Following the successful introduction of two benign solvents for cleaving protected acid peptide fragments from 2‐chlorotrityl chloride (2‐CTC) resin, there is a need to green the cleavage process for obtaining protected peptide amide fragments. In this work, *p*‐xylene and toluene are introduced as greener alternates to dichloromethane (DCM) for preparing protected peptide amide fragments from a Sieber amide resin. The *N‐*dealkylation is a demanding chemical reaction, requiring that the cleavage protocol be optimised to ensure complete cleavage from the resin. After a 30 min reaction time, only 66 % of the final peptide product was retrieved even with the conventional dichloromethane solvent. Therefore, 120 min was considered sufficient to fully cleave the peptide from the Sieber amide resin. This work reaffirms the fact that greening strategies are far from detrimental, with green alternatives often outperforming their replaced counterparts.

## Graphical abstract

***p*** **‐Xylene and toluene** are shown to be greener alternatives to DCM for preparing protected peptide amides from Sieber amide resin for subsequent key reactions such as cyclisation and fragment condensation. Toluene can be considered a universal solvent for cleaving peptides from both 2‐chlorotrityl chloride (2‐CTC) and Sieber amide resins. Its use makes the recycling task easier and helps in implementing a circular economy closed‐loop approach.

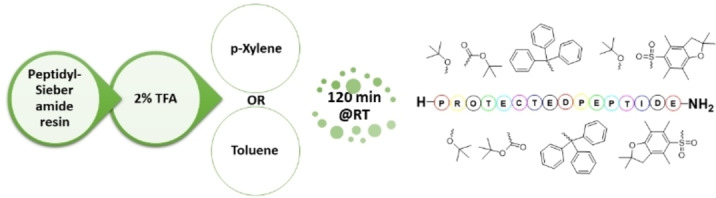

## Introduction

Peptides are consolidating their growing presence in the global pharmaceutical arena. For example, a total of 22 peptides have been approved by the United States Food and Drug Administration (USFDA) over the period from 2017 to 2021.^[1]^ A range of fields have embraced peptides, including immunology,^[2]^ diagnosis,[^3^, ^4^] and drug discovery.[^5^, ^6^] Hence, they are expected to grow from $29 billion to $51 billion between 2020 and 2026.^[7]^ This growth is underpinned by the invention of the solid‐phase peptide synthesis (SPPS) methodology invented by the Nobel Prize laureate Merrifield in 1963.^[8]^ SPPS is the method of choice to prepare peptides on both research and industrial scales.^[9]^ Despite its commercial success, at a production scale, it produces significant levels of solvent and reagent waste.^[10]^ In SPPS, the intermediates are not isolated, and the purification is done by extensive washings through the elongation of the peptide chain.^[11]^ Therefore, solvent consumption is a major concern in SPPS, especially since the solvents used are environmentally unfriendly.[^10^, ^12^, ^13^, ^14^, ^15^] Along with the health and ecosystems concerns, waste solvents have significant negative social and economic consequences that cost many billions per year in the technologically advanced countries.^[7]^

Whilst pharmaceutical innovations will provide huge benefits for the health of humankind, it remains incumbent upon the pharmaceutical industry to ensure that their products are delivered in a sustainable and cost effective manner.^[16]^ Various green alternatives have been introduced to the SPPS process,[^10^, ^17^] aimed at the swelling of the resin,[^15^, ^18^, ^19^, ^20^, ^21^, ^22^, ^23^, ^24^, ^25^, ^26^, ^27^] 9‐fluorenylmethoxycarbonyl (Fmoc) removal,[^12^, ^15^, ^18^, ^20^, ^21^, ^22^, ^24^, ^27^, ^28^, ^29^, ^30^, ^31^] the incorporation of the first amino acid onto the resin,[^32^, ^33^] amide bond formation,[^12^, ^14^, ^15^, ^20^, ^21^, ^22^, ^23^, ^24^, ^25^, ^27^, ^30^, ^34^, ^35^, ^36^, ^37^] and precipitation.[^24^, ^32^, ^38^] Significant advances were achieved in the development of aqueous SPPS (ASPPS) as well.^[39]^

Protected peptide fragments are the essential precursors for key subsequent reactions such as cyclization^[40]^ and fragment condensation.[^41^, ^42^] They can also be utilised to circumvent significant side reactions by introducing peptide fragments rather than the amino acid on its own.^[36]^ Protected peptide fragments are obtained with the aid of low concentrations of trifluoroacetic acid (TFA) usually dissolved in dichloromethane (DCM). However, DCM has serious risks in regard to the human health[^43^, ^44^] as it has been classified as carcinogenic by the Occupational Safety and Health Administration (OSHA)^[45]^ and by the US Environmental Protection Agency (EPA)^[46]^ which both prohibited its use.^[46]^ Greener alternative solvents are introduced in this paper to replace the uses of toxic DCM.

Recently, Albericio's group also has introduced anisole and 1,3‐dimethoxybenzene as green alternatives for replacing DCM for cleaving protected acid peptide fragments from 2‐CTC resin using 2 % (*v*/*v*) TFA in either of these new solvents.^[13]^ It is also important to introduce greener methodologies for cleaving protected peptide amide fragments. Hence, it is proposed here to use 2 % (*v*/*v*) TFA in either toluene or *p*‐xylene as new greener alternatives to replace DCM for cleaving protected peptide amide fragments from Sieber amide resin. Sieber amide resin was introduced in 1987 to prepare protected peptide amides using a mild acidolysis reaction.^[47]^ An optimisation for this new cleavage protocol was implemented to provide a complete 100 % cleavage yield.

## Results and Discussion

Following on from the work done by Albericio's group,^[13]^ experiments were carried out to cleave the protected peptide (Fmoc‐Y(*t*Bu)GL‐NH_2_) from Sieber amide resin (Figure S1). The same reaction protocol developed for the 2‐CTC resin was utilized; where the reaction was done for 30 min using 2 % TFA (*v*/*v*) in DCM, anisole, or toluene. However, our findings confirmed the different behaviour between Sieber amide and 2‐CTC resin in terms of the optimum green solvents as well as the reaction time. Only 66.3 % cleavage yield was observed with the common DCM solvent (Table 1, entry 1; Figure S2).

**Table 1:** Cleaving protected peptide yields following the protocol of 2‐CTC.^[13]^

Exp#	Cleavage solution	Time [min]	Cleaved yield [%]
1	2 % TFA in DCM	30	66.3
2	2 % TFA in anisole	30	10.6
3	2 % TFA in toluene	30	69.4
4	2 % TFA in DCM	60	80.8
5	2 % TFA in DCM	120	94.0

**Figure 1:**
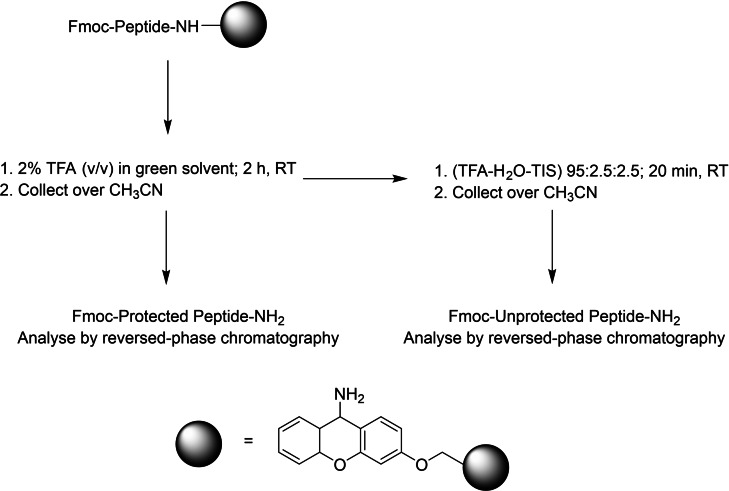
Schematic representation for the cleavage protocol from Sieber amide resin.

Whilst anisole and toluene were able to completely cleave the protected acid peptides from 2‐CTC resin (100 %),^[13]^ they both showed poor performance with Sieber amide resin, where only 10.6 % and 69.4 % of the protected peptide was cleaved, respectively (Table 1, entries 2 and 3; Figures S3 and S4). Given the poor performance of DCM at 30 min reaction time, a decision was made to optimise the cleavage time until a high cleavage yield was achieved. Thus, the reaction time has been extended to 60 and 120 min and the cleavage percentage was monitored by reversed‐phase HPLC. Allowing the reaction to proceed for 60 min has rendered about 80.8 % (Table 1, entry 4; Figure S5) of the protected peptide amide, and about 94 % after 120 min (Table 1, entry 5; Figure S6). Thus, 120 min is considered the optimum reaction time in this work (Figure 1).

### Green solvents screening

Eleven solvents were selected as green/greener alternatives to prepare the cleavage solution; namely, 2‐methyl tetrahydrofuran (2‐MeTHF), *N*‐butyl pyrrolidinone (NBP), γ‐valerolactone (GVL), R‐(+)‐limonene, EtOAc‐CH_3_CN (1 : 1), isoamyl acetate, α‐pinene, *p*‐xylene, cyclohexanone, anisole, and toluene. DCM has been also included as a reference. Solvents were selected as green/greener candidates based on their green attributes, see Table 2.

**Table 2:** Green attributes of solvents used in the study.

Green solvent	Green attribute	Reference
2‐MeTHF^[a]^	Biodegradable, non‐mutagenic, derived from biomass/renewable resources, non‐carcinogenic, and non‐reprotoxic	[48–53]
NBP^[b]^	Biodegradable, non‐mutagenic, non‐reproductively toxic	[54]
GVL^[c]^	Biodegradable, non‐toxic, derived from biomass/renewable resources, non‐carcinogenic, non‐mutagenic	[53, 55]
R‐(+)‐Limonene	Biodegradable, non‐carcinogenic, non‐mutagenic, and non‐reprotoxic	[53, 56]
EtOAc‐CH_3_CN 1 : 1)	(EtOAc): non‐skin irritant, or sensitizer, non‐phototoxic or photo allergenic CH_3_CN: non‐mutagenic, non‐toxic	[57, 58]
Isoamyl acetate	Flavouring component, non‐carcinogenic, non‐mutagenic, and non‐reprotoxic	[53, 59]
α‐Pinene	Renewable bio‐solvent, non‐carcinogenic, non‐mutagenic, and non‐reprotoxic	[53, 60]
*p*‐Xylene	Renewable, non‐carcinogenic, non‐mutagenic, and non‐reprotoxic	[53, 61]
Cyclohexanone	Non‐carcinogenic, non‐mutagenic, and non‐reprotoxic	[53]
Anisole	Biodegradable, low‐cost, non‐toxic.	[62]
Toluene	Non‐carcinogenic, non‐mutagenic	[63]

[a] 2‐methyl tetrahydrofuran: 2‐MeTHF; [b] NBP: *N*‐butyl pyrrolidinone; [c] GVL: γ‐valerolactone.

Green solvents were screened using the following 5‐mer peptide Fmoc‐Y(*t*Bu)GFGL‐NH_2_ (Figure S7). The peptidyl resin was treated with the cleavage solution containing 2 % TFA (*v*/*v*) in the green solvent and including DCM as a control, independently. The filtrate was collected over CH_3_CN and monitored using HPLC. The same peptidyl resin was treated with high percentage of TFA‐H_2_O‐triisopropyl silane (TIS) (95 : 2.5 : 2.5) to evaluate the extent of the cleavage and wether the peptide was completely cleaved from the resin or not (Figures S8‐S30). The results in Table 3 demonstrate the performance of each cleavage solution.

**Table 3:** Green solvents screening for cleaving protected peptide amide fragments from Sieber amide resin.

Exp#	Cleavage solution	Cleaved yield [%]
1	DCM	99.0
2	2‐MeTHF^[a]^	3.9
3	NBP^[b]^	2.9
4	GVL^[c]^	3.7
5	R‐(+)‐Limonene	4.2
6	EtOAc‐CH_3_CN (1 : 1)	3.5
7	Isoamyl acetate	4.3
8	α‐Pinene	4.7
9	*p*‐Xylene	90.2
10	Cyclohexanone	18.4
11	Anisole	8.8
12	Toluene	93.5

[a] 2‐methyl tetrahydrofuran: 2‐MeTHF; [b] NBP: *N*‐butyl pyrrolidinone; [c] GVL: γ‐valerolactone.

*p*‐Xylene and toluene are able to cleave the protected peptide with an acceptable yield 90.2 % and 93.5 % (Table 3, entries 9 and 12; Figures S14 and S17), respectively, versus 99 % in case of DCM (Table 3, entry 1; Figure S8). Both solvents are classified in the yellow region according to the GSK solvent sustainability guide,^[64]^ and as usable according to the Pfizer guide.^[65]^ They are not the optimum solvents in terms of their greenness, but still more preferable and greener than DCM. The higher boiling points of *p*‐xylene (138 °C)^[53]^ and toluene (111 °C),^[53]^ in comparison to that of DCM (40 °C),^[53]^ means that no premature evaporation will take place during the synthesis. On the other hand, this phenomenon is common in the case of DCM, and it leads to the removal of acid labile groups (e. g., Trt), as a result of increasing the TFA concentration.^[13]^ Anisole has been confirmed to be unsuitable for cleaving protected peptides from Sieber amide resin (Table 3, entry 11).

To examine the suitability of the chosen conditions, two dipeptides containing the most acid‐labile sidechain protecting group, the trityl (Trt) group, were synthesised: H−H(Trt)L−NH_2_ and H−N(Trt)L−NH_2_. The two peptidyl resins were treated with the cleavage solution of 2 % TFA (*v*/*v*) in *p*‐xylene, toluene, and DCM, independently. The optimised protocol was followed as shown in Figure 1. No evidence of Trt group removal was observed in both peptides, which confirms the suitability of the optimised cleavage conditions in this study (Figures S31–S38).

Finally, three therapeutic peptides were selected to demonstrate the performance of the new cleavage protocol. The selection considered peptides that are synthesised following the fragment condensation approach. Hence, the fragment that is anchored to the resin will be synthesised and examined against our protocol. (i) Abaloparatide is a 34‐mer peptide developed by the biotech company Radius Health, Inc (Waltham, MA, USA) (Figures S39–S42). It selectively activates the parathyroid hormone 1 receptor. Abaloparatide received USFDA approval in 2017.^[3]^ (ii) Aviptadil is 28‐mer synthetic Vasoactive Intestinal Peptide (VIP) that is being developed to treat patients with critical COVID‐19 respiratory failure (Figures S43–S46).^[66]^ (iii) Enfuvirtide (T‐20 or Fuzeon) is a 36‐mer membrane fusion inhibitor for the treatment of HIV, which was approved by the USFDA in 2003 (Figures S47–S50).^[67]^

**Figure 2:**
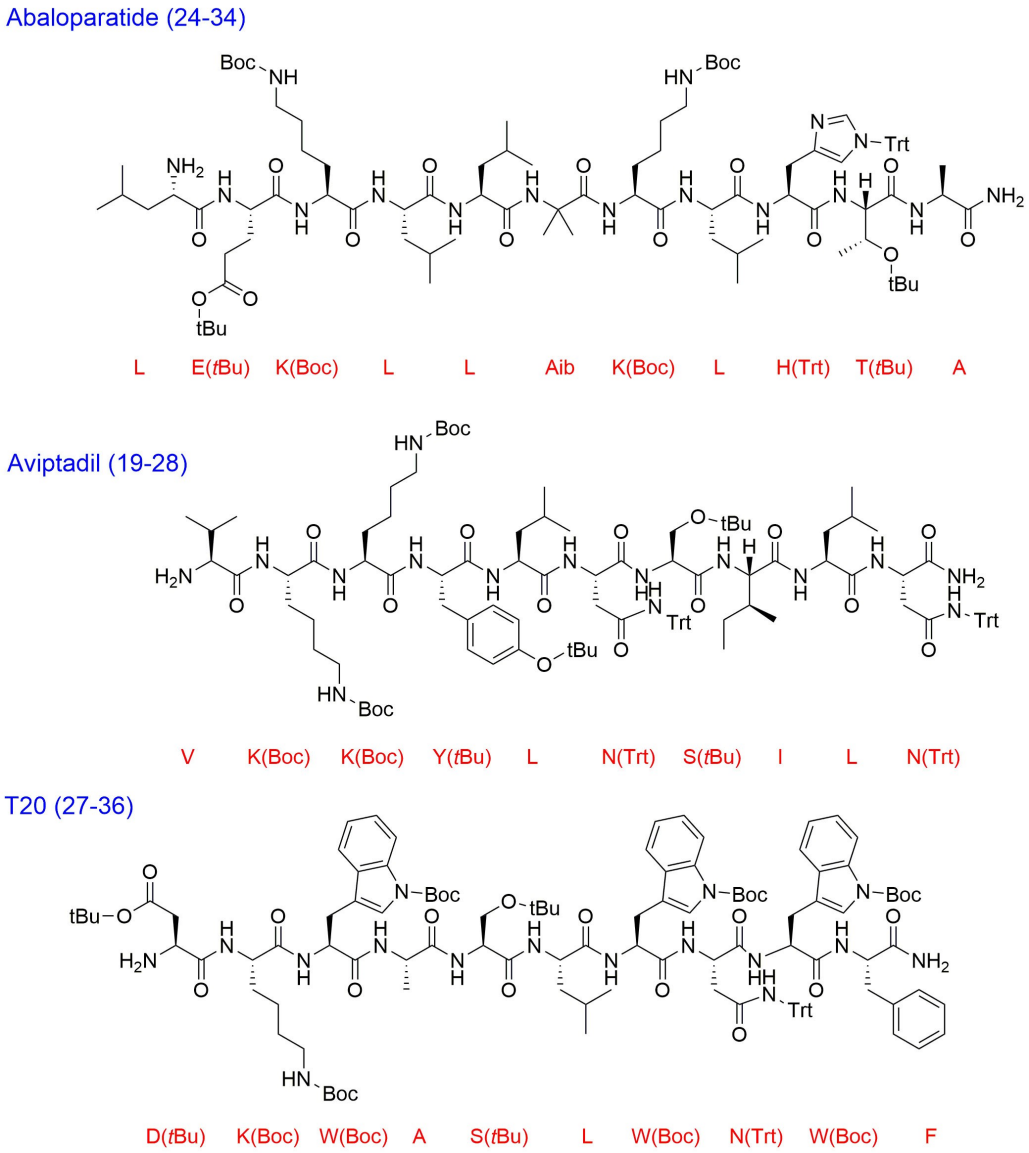
Chemical structures of the peptide fragments tested in this study. Boc: *tert*‐butyloxycarbonyl; *t*Bu: *tertiary*‐butyl; Trt: trityl; Aib: 2‐aminoisobutyric acid.

From either the published research and/or filed patents, the following fragments were identified to be synthesised on the Sieber amide resin; (i) Abaloparatide, fragment (24–34) [LEKLLAibKLHTA],^[68]^ (ii) Aviptadil, fragment (19–28) [VKKYLNSILN],^[69]^ (iii) Enfuvirtide, fragment (27–36) [DKWASLWNWF] (Figure 2).^[67]^

**Figure 3:**
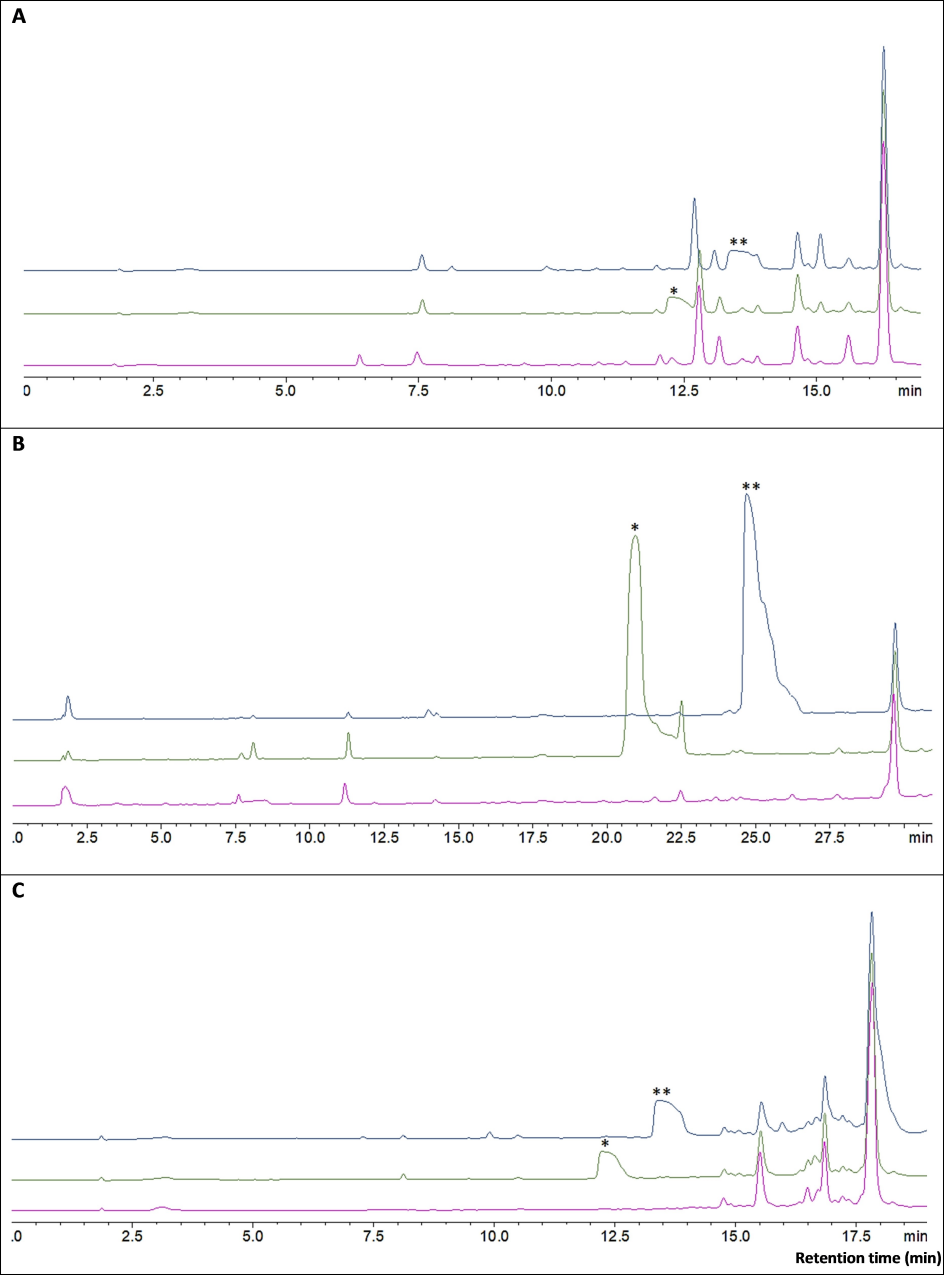
Protected peptides: **A** Fmoc‐Abaloparatide fragment (24‐34): Fmoc‐LE(*t*Bu)K(Boc)LLAibK(Boc)LH(Trt)T(*t*Bu)A‐NH_2_. **B**. Aviptadil fragment (19–28): H‐VK(Boc)K(Boc)Y(*t*Bu)LN(Trt)S(*t*Bu)ILN(Trt)‐NH_2_. **C**. T20 fragment (27–36): H−D(*t*Bu)K(Boc)W(Boc)AS(*t*Bu)LW(Boc)N(Trt)W(Boc)F‐NH_2_. Pink trace: 2 % TFA in DCM; green trace: 2 % TFA in toluene; blue trace: 2 % TFA in *p*‐xylene (Reaction time 120 min). 5–95 % in 15 min gradient elution for Abaloparatide and T20, where 15–70 % in 30 min for Aviptadil fragment. *λ*=300 nm. Mobile phase A: 0.1 % TFA in H_2_O; mobile phase B: 0.1 % TFA in CH_3_CN; Symmetry Luna C_18_ (3.6 μm, 4.6×150 mm) column. *Toluene peak. **p‐Xylene peak.

These fragments were then cleaved using 2 % TFA in *p*‐xylene, toluene, and DCM as a control. Furthermore, the same resin was treated with TFA‐TIS‐H_2_O (95 : 2.5 : 2.5) to evaluate the extent of the cleavage and whether the whole peptide was cleaved or not. The same purity profile of the cleaved peptides was obtained in all solvents (Figure 3; Table 4), thus confirming the comparable cleavage efficiency in the new greener alternatives with that in conventional DCM.

**Table 4:** Recovered amounts and purities of the cleaved peptide fragments.

Peptide fragment	Cleavage solvent; 2 % TFA[*v*/*v*] in:				
	DCM		*p*‐Xylene		Toluene	
	Cleavage yield [%]	Purity [%]	Cleavage yield [%]	Purity ([%]	Cleavage yield [%]	Purity [%]
Abaloparatide (24–34)	41.7	58.3	52.2	62.5	43.1	69.8
Aviptadil (19–28)	42.4	86.9	43.1	96.2	48.4	85.4
Enfuvirtide T‐20 (27–36)	43.1	75.7	41.2	85.4	49.2	78.2

While assessing the extent of the cleavage by treating the resin with 95 % of TFA, a cleavage of the Sieber amide linker from the resin was observed. This is in line with the fact that side reactions do take place through the ether bond that anchors the linker to the resin.^[70]^ The observed phenomenon increases proportionally with time (Figure S51). Hence, for assessing the cleavage extent, the resins were treated with TFA‐TIS‐H_2_O (95 : 2.5 : 2.5) for 20 min only. No traces of the peptide were observed after treating the resin with 95 % TFA. Therefore, this proves the efficiency of the optimised protocol to fully cleave the peptide from the resin (Figures S51–S61).

Due to an observed overlap between the Abaloparatide fragment and the peak due to *p*‐xylene, the Fmoc‐protected peptide was analysed instead. Furthermore, as the Fmoc‐protected fragments were considered during the optimisation steps, we have also carried out additional experiments on the Fmoc‐protected peptides of Aviptadil and T20 to further investigate the optimised protocol (Figures S62–S68). To compare the recovered yields and purities of the cleaved peptide fragments in the three solvents, the cleaved peptide solutions were collected over water, and then the solutions were submitted to freeze‐drying. The recovered peptides amounts were as follows (Table 4):

As shown in Table 4, comparable yields and purities of the peptides in either of the tested solvents were obtained. It is worth highlighting the higher cleavage yields of all peptides when cleaving with the greener alternative solvents versus DCM.

## Conclusion

Exhaustive screening of eleven green solvents has shown that *p*‐xylene and toluene emerged as greener alternatives to DCM to cleave protected peptide amide fragments from Sieber amide resin. Toluene is considered a versatile greener alternate to cleave both protected acid peptides and protected peptide amide fragments from 2‐CTC^[13]^ and Sieber amide resin, respectively. Hence, from a circular economy point of view, toluene shall be considered as a universal solvent for this purpose. However, longer reaction times are required to quantitively cleave the peptide fragment from Sieber amide resin. While 30 min were enough when cleaving from 2‐CTC resin,^[13]^ up to 120 min were required to fully cleave the peptide fragments from Sieber amide resin. Such phenomenon was previously shown in Albericio's group which confirmed that longer reaction time (120 min) is required to quantitatively cleave peptides from rink amide resin.^[71]^ N‐xanthen and N‐benzhydryl are the N−C bonds where the reaction takes place to cleave the peptide from Sieber and Rink amide resins, respectively. This *N*‐alkyl bond is generally stable and comprises a high dissociation energy, which explains the difficulty in its cleavage.[^72^, ^73^]

It is to be emphasised that the longer reaction time did not alter the final chemical structure of the protected peptide. The stability of the most acid‐labile sidechain protecting group (Trt) was demonstrated. Cleaving H−H(Trt)L−NH_2_ and H−N(Trt)L−NH_2_ peptides following the protocol in this study showed no evidence of Trt group removal with the greener alternates nor with DCM. Comparable purities and higher yields of the cleaved peptides were obtained when cleaving in the greener alternatives versus DCM. We thus envisage that *p‐*xylene and toluene will replace DCM for cleaving protected peptide amide for subsequent key reactions both at research and industrial settings.

## Experimental Section

### Materials and Methods

Merck Sieber amide (0.75 mmol g^−1^, supplier‘s specification) resins were used for all syntheses. All reagents and solvents were obtained from commercial suppliers and were used without further purification unless otherwise stated. Analytical HPLC was performed on Shimadzu LC20 system using Lab solution software for data processing. Column: Symmetry Luna C18 (3.6 μm, 4.6×150 mm) column, with flow rate of 1.0 mL min^−1^ and UV detection at 220 & 300 nm. Mobile phase A was 0.1 % TFA in H_2_O, and mobile phase B was 0.1 % TFA in CH_3_CN. The mass spectrometry of the peptide fragments was performed on a Velos Pro (ThermoFisher Scientific), a hybrid linear trap quadrupole (LTQ)‐Orbitrapmass spectrometer. The samples were directly infused into the system.

### Peptide Synthesis

Peptides were synthesised following the standard methodology performed in our laboratory (3 equiv. of Fmoc‐AA‐OH, 3 equiv. of OxymaPure, 3 equiv. of *N,N’*‐diisopropylcarbodiimide (DIC)) in *N,N*‐dimethylformamide (DMF) and then shaking for 30 min. Fmoc was removed using 20 % piperidine/DMF and the mixture was allowed to shake for 2 and 7 min. The Fmoc‐amino acids (3 equiv.) and OxymaPure (3 equiv.) were dissolved in a minimum amount of DMF (0.5 mL/100 mg resin) and sonicated for 10 min. DIC (3 equiv.) was then added to the solution, which in turn was added to the previously swelled resin and allowed to react for 30 min under mechanical shaking. Finally, the resin was washed twice with DCM and dried under vacuum.

### Cleavage Protocols

#### Protected fragments

Peptide resin was placed in a syringe fitted with porous polyethylene filter. It was then swelled with the corresponding solvent for 10 min. The solvent was then filtered off, the cleavage solution (2 mL, 2 % TFA (*v*/*v*) in the greener alternates) was added per 100 mg of the peptide resin, and the syringe was closed with a cap and shaken for 120 min at rt. Finally, the filtrate was collected over CH_3_CN (4.0 mL), and the cleaved resin was washed 4 times with CH_3_CN (1 mL each time). The filtrates were again collected in the same tube. An aliquot (5 μL) was then injected into the HPLC system. Finally, to check the efficiency of the cleavage protocol, the resin was dried and treated with TFA‐TIS‐H_2_O (95 : 2.5 : 2.5) (600 μL). The same work‐up was followed, and the resulting solution was monitored by HPLC.

#### Unprotected fragments

The final synthesised peptide was cleaved from the resin using TFA/TIS/H_2_O (95 : 2.5 : 2.5) (1 mL/100 mg) under mechanical shaking for 20 min. Chilled diethyl ether was then added (5 times the cleavage solution volume), and the solution was kept in an ice bath for 30 min. The solution was then centrifuged for 5 min at 5000 rpm, and the supernatant was decanted. A new amount of the ether (5 times the cleavage solution volume) was added to repeat this step. Any remaining ether was dried under N_2_. Finally, the precipitate was dissolved in CH_3_CN−H_2_O (1 : 1). A small amount of the solution was injected into HPLC system to check the purity of the final product.

#### Cleavage yield determination

The same protocol given for the protected fragments was followed. However, the filtrate was collected over cold water (5 times the cleavage solution volume), and then the solution was submitted to freeze‐drying using a lyophilizer machine. The % yield was calculated with respect to the scale of synthesis accordingly.

## Supporting Information Summary

The Supporting Information contains HPLC chromatograms and mass spectra of the synthesised peptides.

## Conflict of interest

The authors declare no conflict of interests.

1

## Supporting information

As a service to our authors and readers, this journal provides supporting information supplied by the authors. Such materials are peer reviewed and may be re‐organized for online delivery, but are not copy‐edited or typeset. Technical support issues arising from supporting information (other than missing files) should be addressed to the authors.

Supporting Information

## Data Availability

The data that support the findings of this study are available on request from the corresponding author. The data are not publicly available due to privacy or ethical restrictions.
