# Supplementary material for: Greener Cleavage of Protected Peptide Fragments from Sieber Amide Resin
Source: ChemistryOpen. 2022 Dec 23;11(12):e202200236. doi: 10.1002/open.202200236 (PMC9789020; doi:10.1002/open.202200236)
Supplement: Supplementary file 1 — Supporting Information [file OPEN-11-e202200236-s001.pdf]

ChemistryOpen

Supporting Information

Greener Cleavage of Protected Peptide Fragments from Sieber Amide Resin

Othman Al Musaimi,* Varshitha Gavva, and Daryl R. Williams

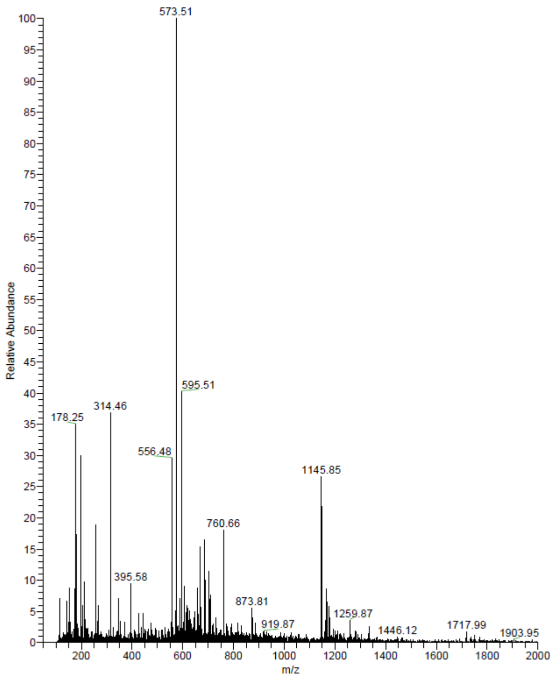

Supplementary Figure 1. Mass of Fmoc-YGL-NH_2. Calculated: 572.66; found: 573.51 $[M+H]^+$, 1145.85 $[2M+H]^+$ (non-covalent dimer).

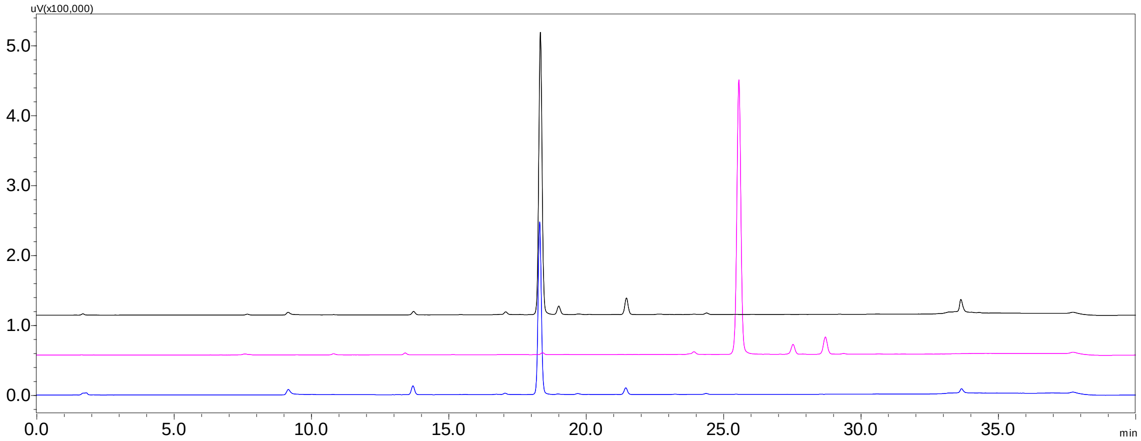

Supplementary Figure 2. Cleaved Fmoc-Y(*t*Bu)GL-NH_2. Blue: 2%TFA in DCM + 95% TFA; Pink: 2%TFA in DCM; Black: Fmoc-YGL-NH_2 as reference. (Reaction time 30 min). 15–70% in 30 min gradient elution. λ = 300 nm. Mobile phase A: 0.1% TFA in H_2O; mobile phase B: 0.1% TFA in CH_3CN; Symmetry Luna C18 (3.6 μm, 4.6 × 150 mm) column.

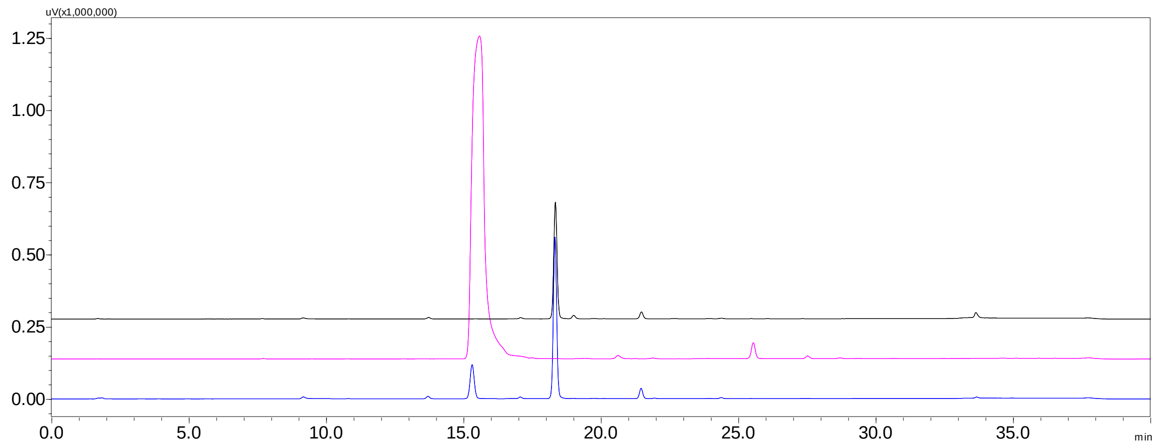

Supplementary Figure 3. Cleaved Fmoc-Y(*t*Bu)GL-NH_2. Blue: 2%TFA in anisole + 95% TFA; Pink: 2%TFA in anisole; Black: Fmoc-YGL-NH_2 as reference. (Reaction time 30 min). Refer for legend of Fig. S2 for chromatographic conditions.

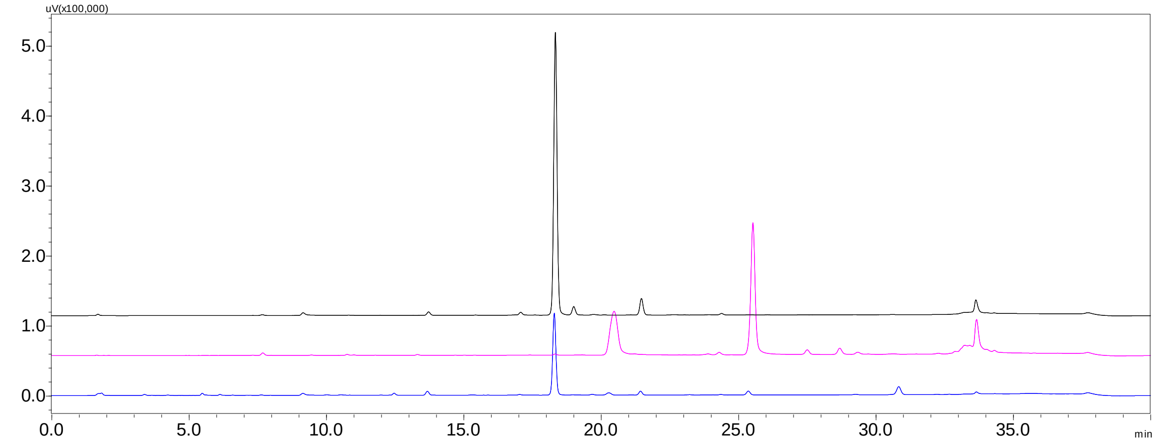

Supplementary Figure 4. Cleaved Fmoc-Y(*t*Bu)GL-NH_2. Blue: 2%TFA in toluene + 95% TFA; Pink: 2%TFA in toluene; Black: Fmoc-YGL-NH_2 as reference. (Reaction time 30 min). Refer for legend of Fig. S2 for chromatographic conditions.

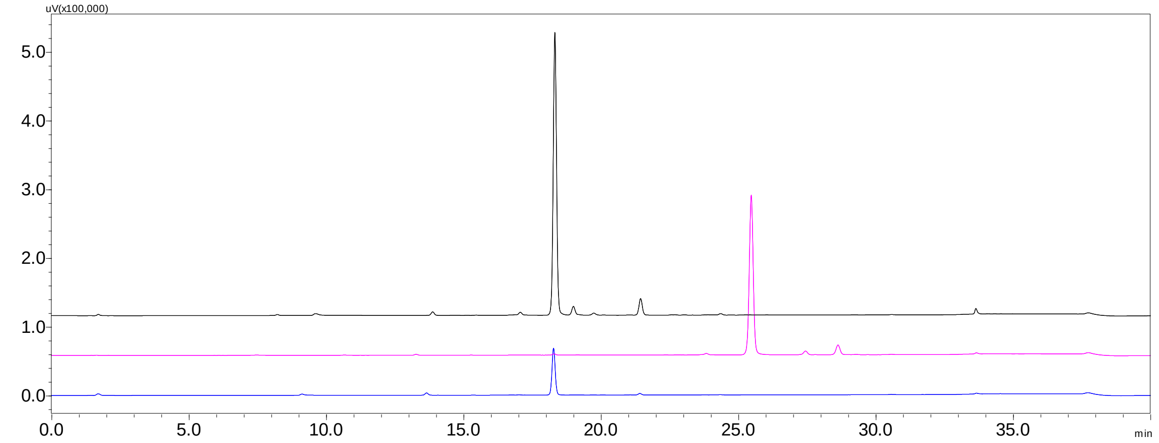

Supplementary Figure 5. Cleaved Fmoc-Y(*t*Bu)GL-NH_2. Blue: 2%TFA in DCM + 95% TFA; Pink: 2%TFA in DCM; Black: Fmoc-YGL-NH_2 as reference. (Reaction time 60 min). Refer for legend of Fig. S2 for chromatographic conditions.

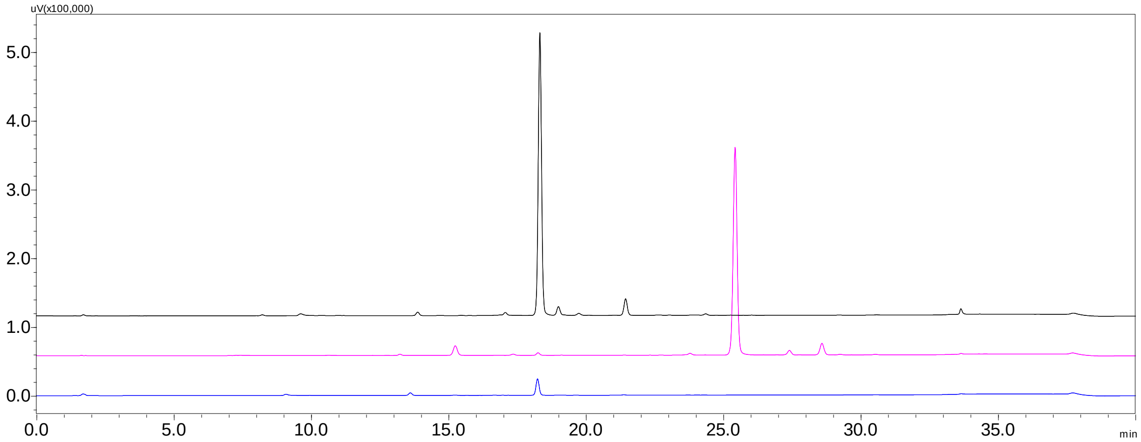

Supplementary Figure 6. Cleaved Y(*t*Bu)GL-NH_2. Blue: 2%TFA in DCM + 95% TFA; Pink: 2%TFA in DCM; Black: Fmoc-YGL-NH_2 as reference. (Reaction time 120 min). Refer for legend of Fig. S2 for chromatographic conditions.

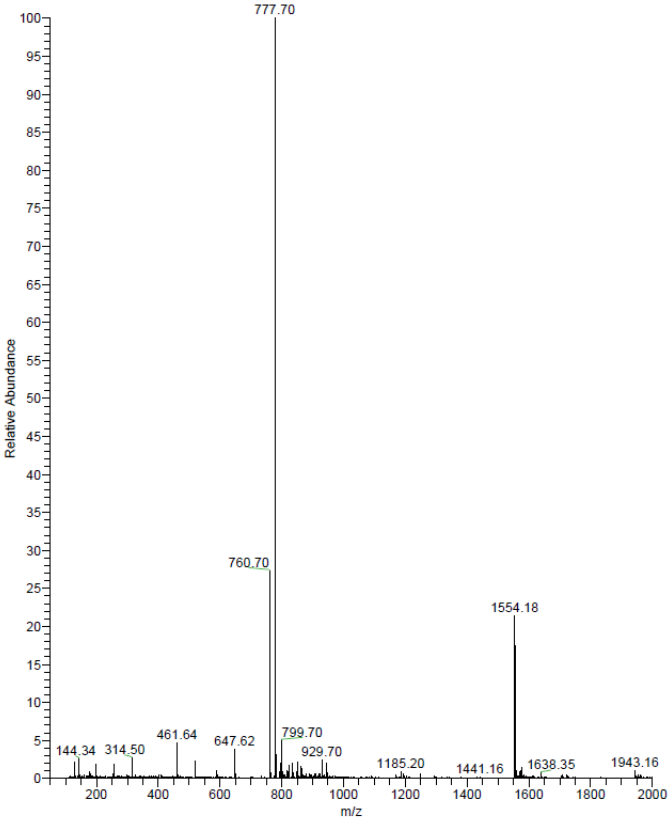

Supplementary Figure 7. Mass of Fmoc-YGFGL-NH_2. Calculated: 776.34, found: 777.70 $[M+H]^+$, 1554.18 $[2M+H]^+$ (non-covalent dimer), 760.70.18 $[M-17+H]^+$

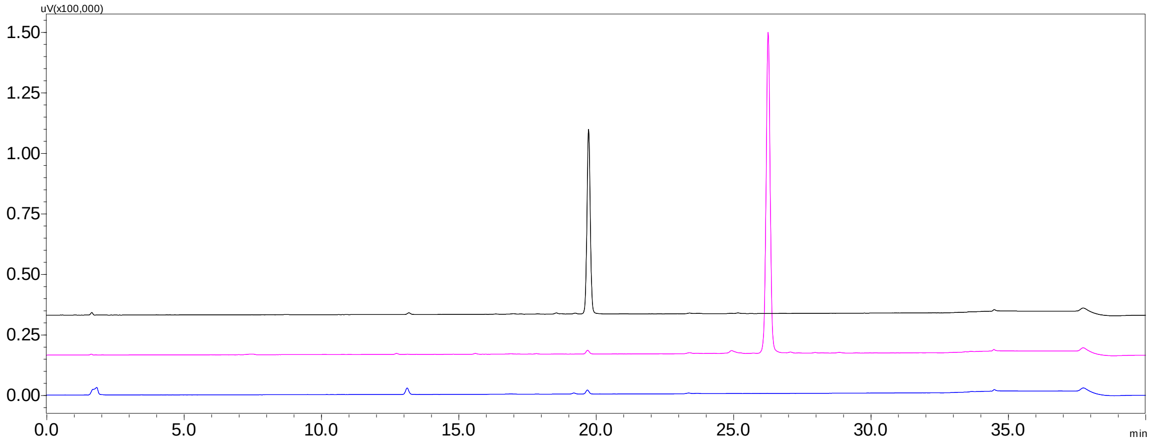

Supplementary Figure 8. Cleaved Fmoc-Y(*t*Bu)GFGL-NH_2. Blue: 2%TFA in DCM + 95% TFA; Pink: 2%TFA in DCM; Black: Fmoc-YGFGL-NH_2 as reference. (Reaction time 120 min). Refer for legend of Fig. S2 for chromatographic conditions.

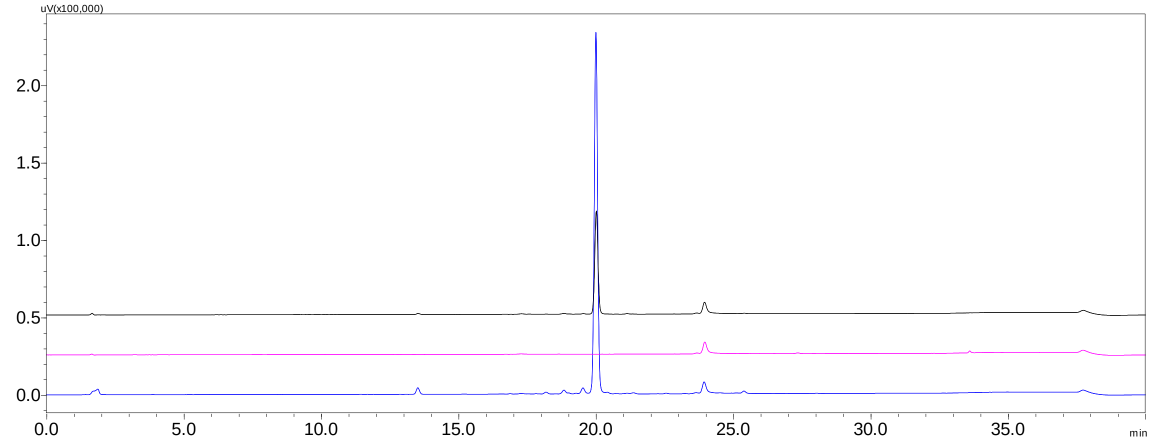

Supplementary Figure 9. Cleaved Fmoc-Y(tBu)GFGL-NH2. Blue: 2%TFA in 2-MeTHF + 95% TFA; Pink: 2%TFA in 2-MeTHF; Black: Fmoc-YGFGL-NH_2 as reference. (Reaction time 120 min). Refer for legend of Fig. S2 for chromatographic conditions.

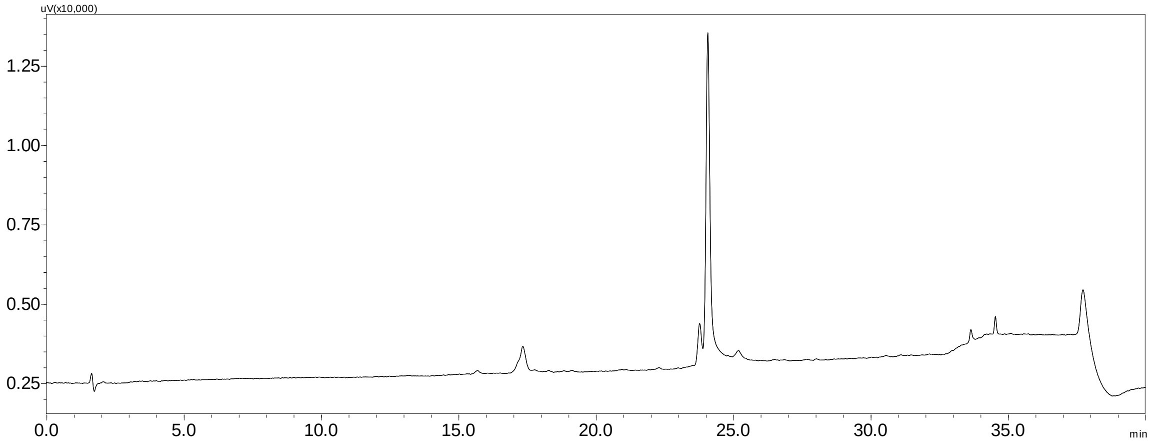

Supplementary Figure 10. Blank injection of 2-MeTHF. Refer for legend of Fig. S2 for chromatographic conditions.

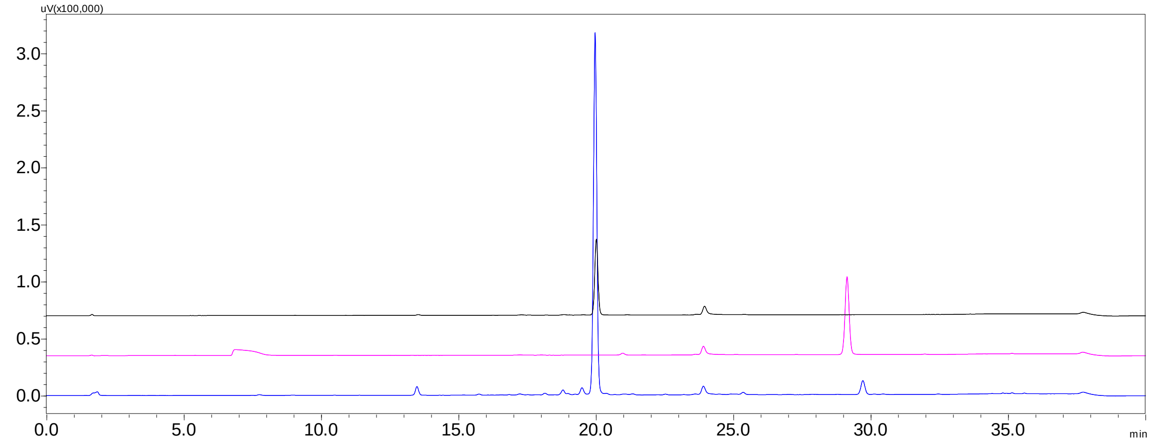

Supplementary Figure 11. Cleaved Fmoc-Y(tBu)GFGL-NH2. Blue: 2%TFA in NBP + 95% TFA; Pink: 2%TFA in NBP; Black: Fmoc-YGFGL-NH_2 as reference. (Reaction time 120 min). Refer for legend of Fig. S2 for chromatographic conditions.

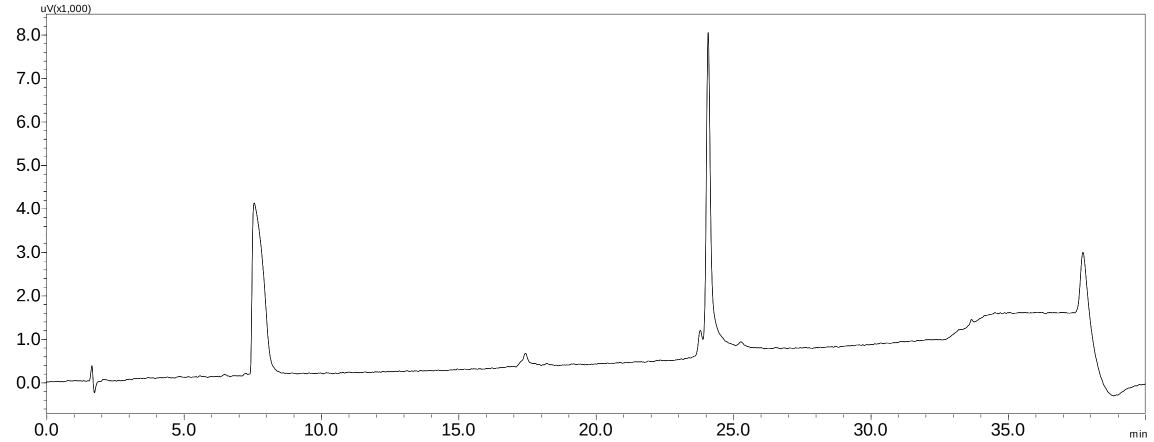

Supplementary Figure 12. Blank injection of NBP. Refer for legend of Fig. S2 for chromatographic conditions.

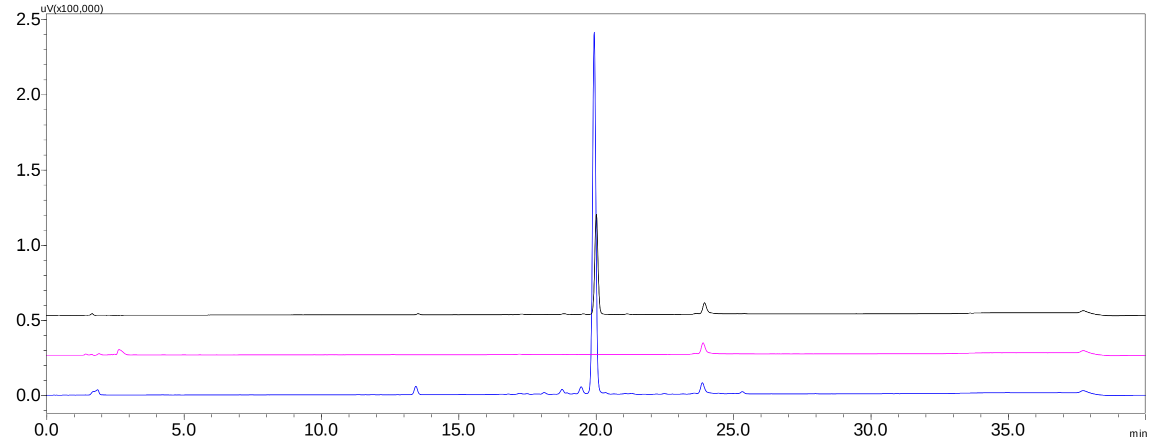

Supplementary Figure 13. Cleaved Fmoc-Y(tBu)GFGL-NH2. Blue: 2%TFA in GVL + 95% TFA; Pink: 2%TFA in GVL; Black: Fmoc-YGFGL-NH_2 as reference. (Reaction time 120 min). Refer for legend of Fig. S2 for chromatographic conditions.

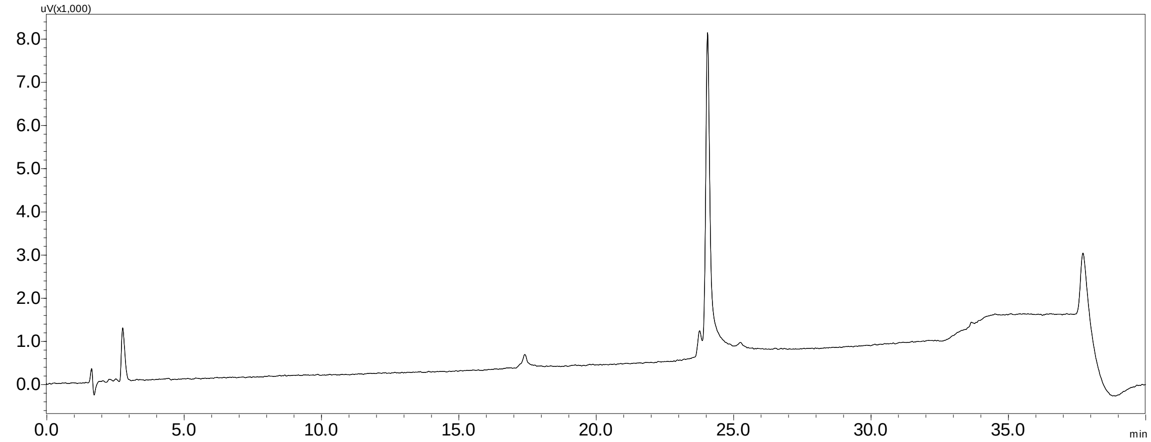

Supplementary Figure 14. Blank injection of GVL. Refer for legend of Fig. S2 for chromatographic conditions.

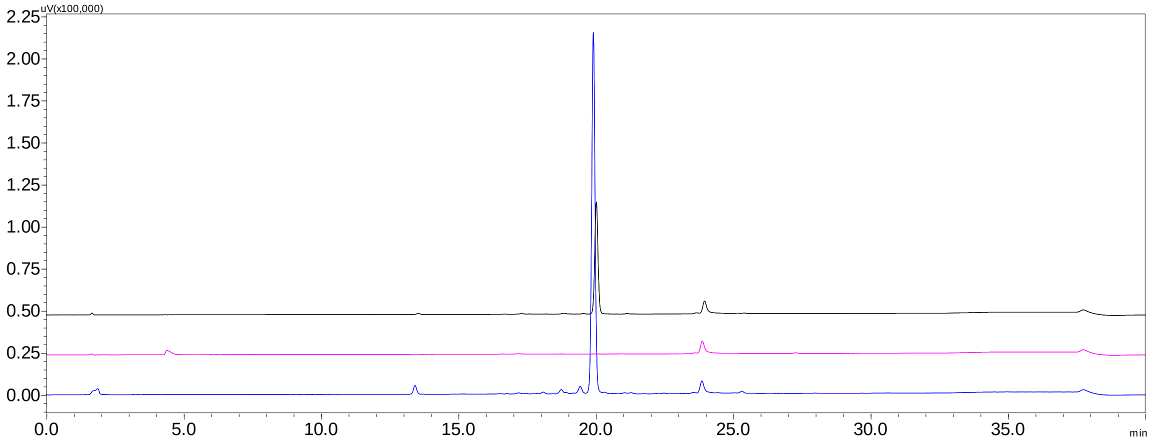

Supplementary Figure 15. Cleaved Fmoc-Y(tBu)GFGL-NH2. Blue: 2%TFA in EtOAc:ACN (1:1) + 95% TFA; Pink: 2%TFA in EtOAc:ACN (1:1); Black: Fmoc-YGFGL-NH_2 as reference. (Reaction time 120 min). Refer for legend of Fig. S2 for chromatographic conditions.

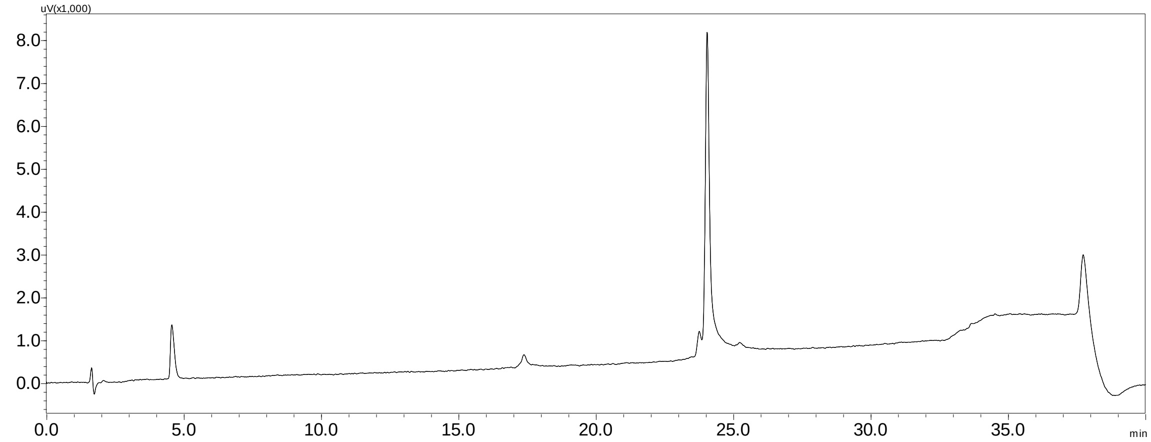

Supplementary Figure 16. Blank injection of EtOAc:ACN (1:1). Refer for legend of Fig. S2 for chromatographic conditions.

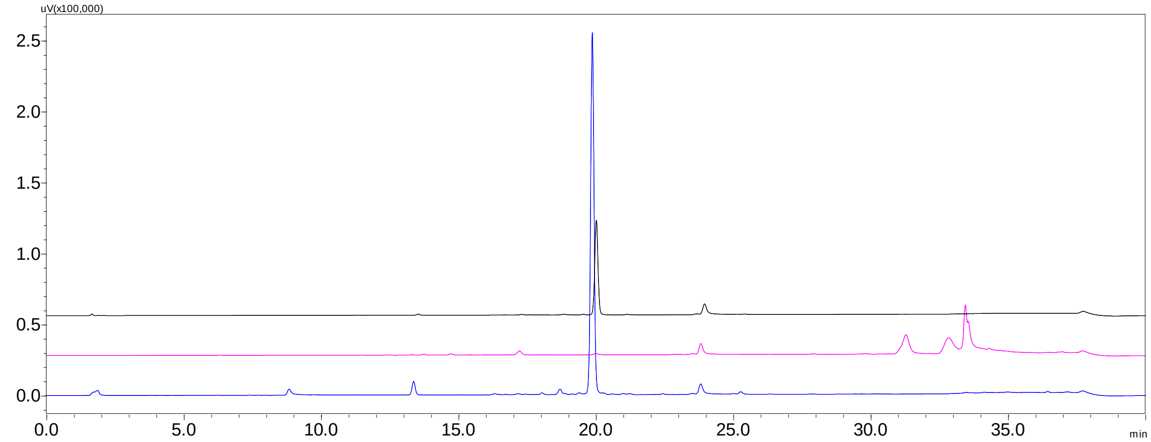

Supplementary Figure 17. Cleaved Fmoc-Y(tBu)GFGL-NH2. Blue: 2%TFA in R-(+) Limonene + 95% TFA; Pink: 2%TFA in R-(+) Limonene; Black: Fmoc-YGFGL-NH_2 as reference. (Reaction time 120 min). Refer for legend of Fig. S2 for chromatographic conditions.

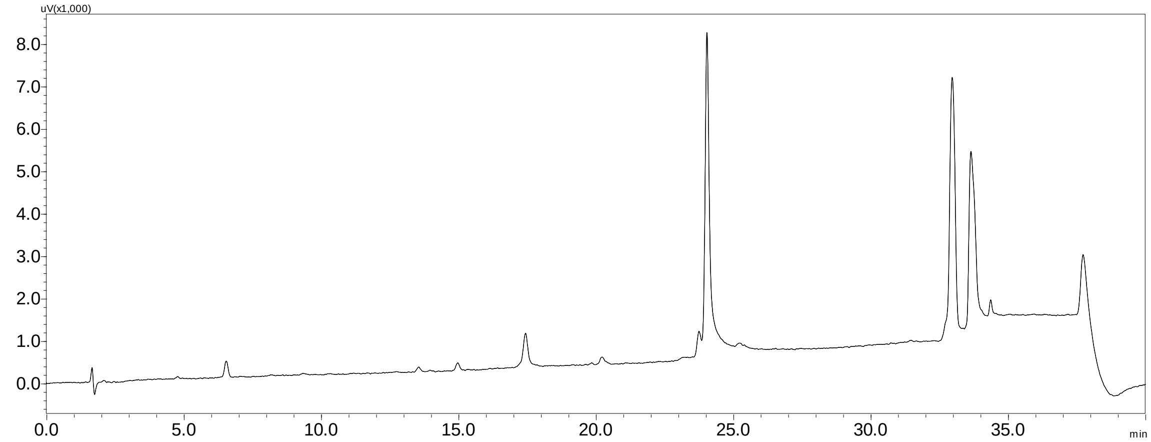

Supplementary Figure 18. Blank injection of R-(+) Limonene. Refer for legend of Fig. S2 for chromatographic conditions.

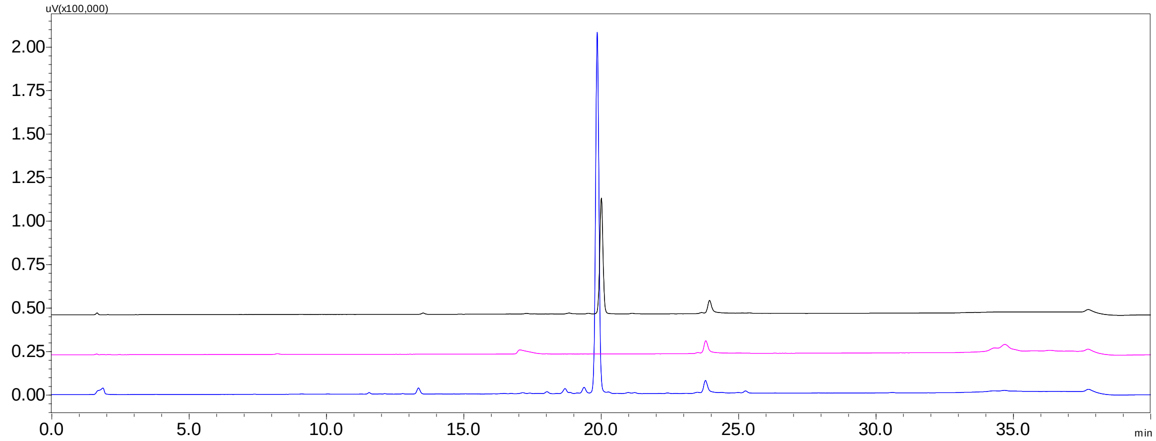

Supplementary Figure 19. Cleaved Fmoc-Y(tBu)GFGL-NH2. Blue: 2%TFA in isoamyl acetate + 95% TFA; Pink: 2%TFA in isoamyl acetate; Black: Fmoc-YGFGL-NH_2 as reference. (Reaction time 120 min). Refer for legend of Fig. S2 for chromatographic conditions.

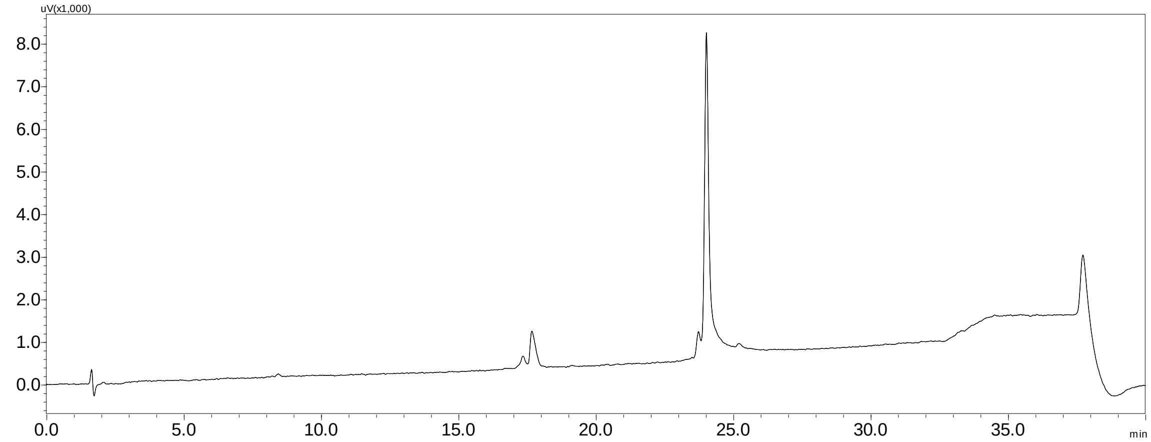

Supplementary Figure 20. Blank injection of isoamyl acetate. Refer for legend of Fig. S2 for chromatographic conditions.

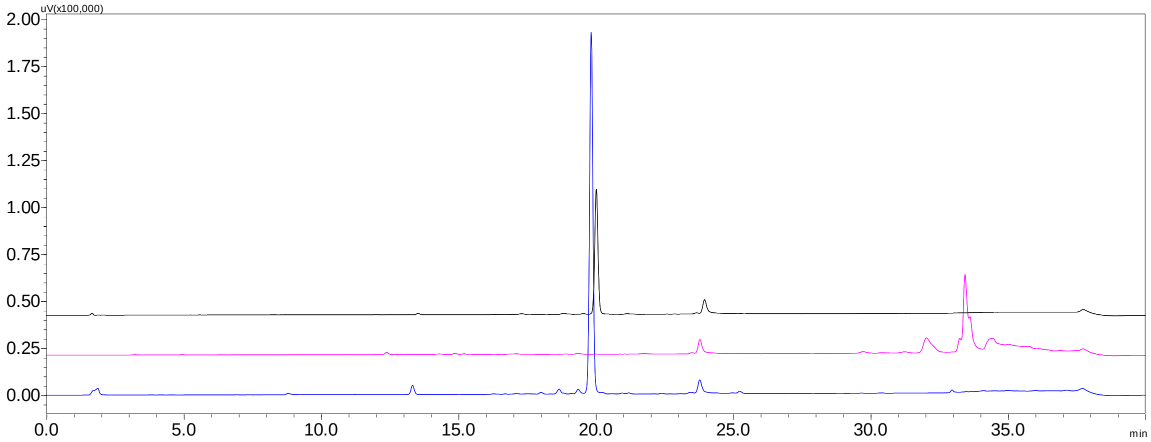

Supplementary Figure 21. Cleaved Fmoc-Y(tBu)GFGL-NH2. Blue: 2%TFA in α-Pinene + 95% TFA; Pink: 2%TFA in α-Pinene; Black: Fmoc-YGFGL-NH_2 as reference. (Reaction time 120 min). Refer for legend of Fig. S2 for chromatographic conditions.

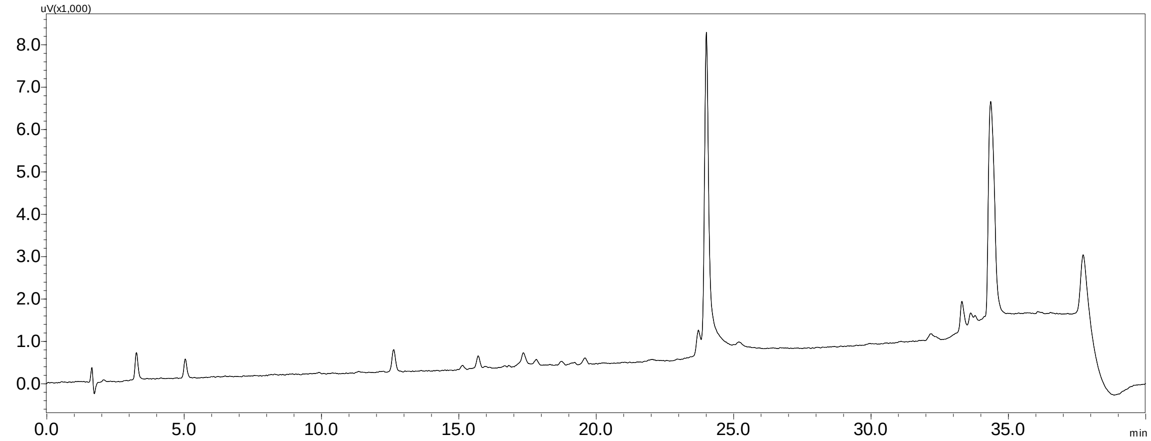

Supplementary Figure 22. Blank injection of α-Pinene. Refer for legend of Fig. S2 for chromatographic conditions.

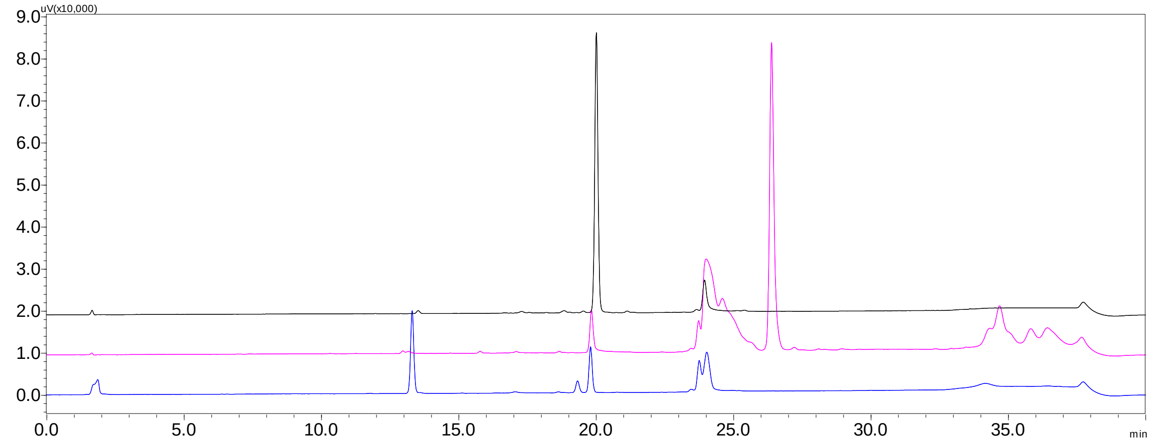

Supplementary Figure 23. Cleaved Fmoc-Y(tBu)GFGL-NH2. Blue: 2%TFA in p-xylene + 95% TFA; Pink: 2%TFA in p-xylene; Black: Fmoc-YGFGL-NH_2 as reference. (Reaction time 120 min). Refer for legend of Fig. S2 for chromatographic conditions.

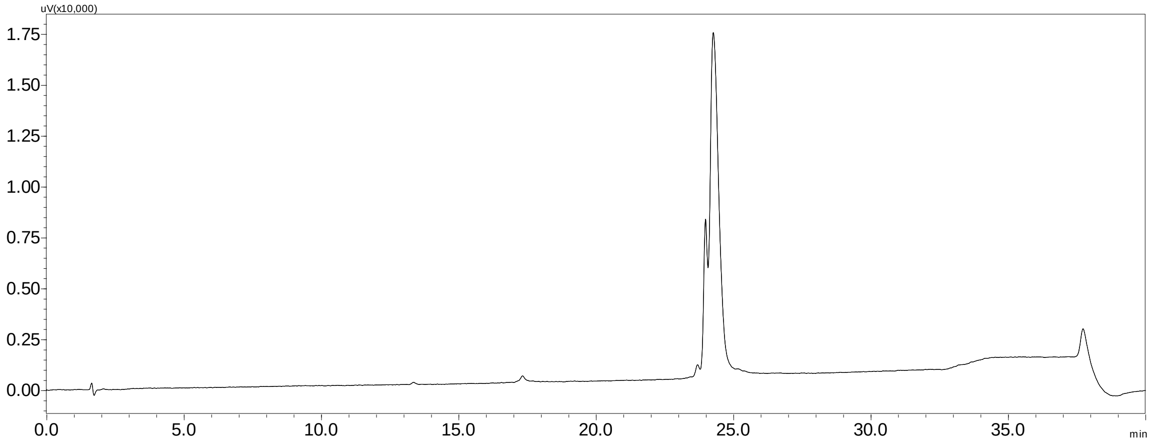

Supplementary Figure 24. Blank injection of p-xylene. Refer for legend of Fig. S2 for chromatographic conditions.

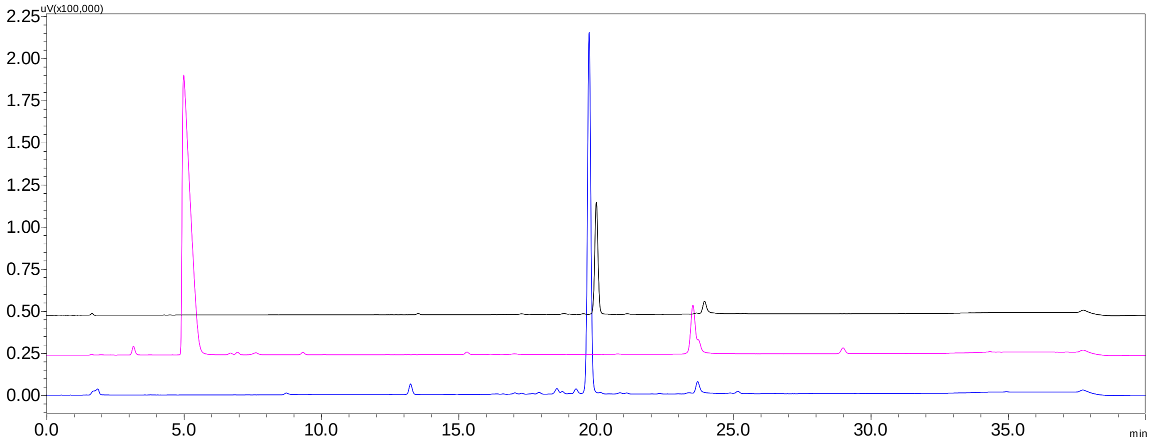

Supplementary Figure 25. Cleaved Fmoc-Y(tBu)GFGL-NH2. Blue: 2%TFA in cyclohexanone + 95% TFA; Pink: 2%TFA in cyclohexanone; Black: Fmoc-YGFGL-NH_2 as reference. (Reaction time 120 min). Refer for legend of Fig. S2 for chromatographic conditions.

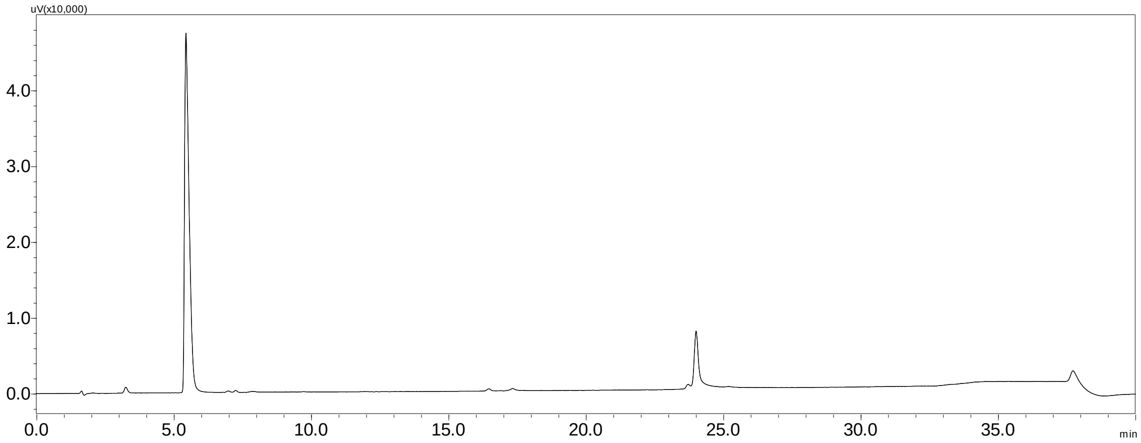

Supplementary Figure 26. Blank injection of cyclohexanone. Refer for legend of Fig. S2 for chromatographic conditions.

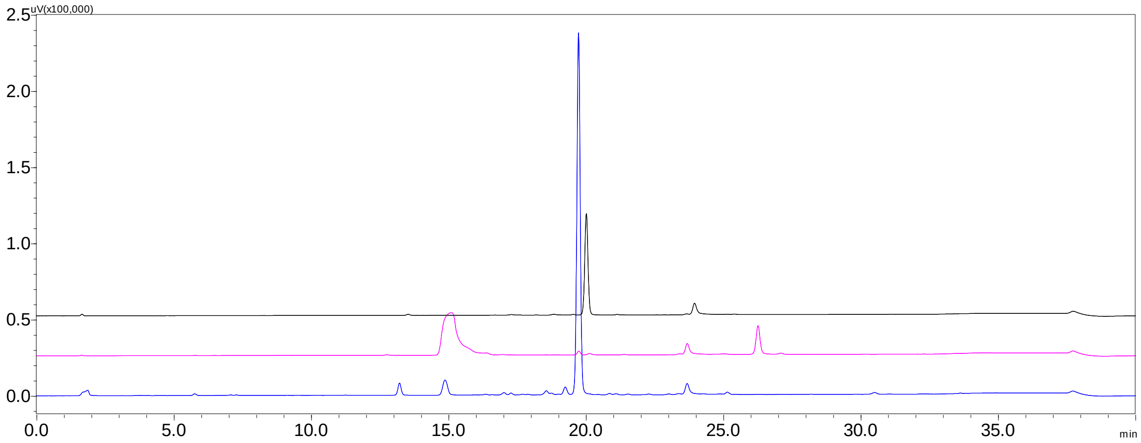

Supplementary Figure 27. Cleaved Fmoc-Y(tBu)GFGL-NH2. Blue: 2%TFA in anisole + 95% TFA; Pink: 2%TFA in anisole; Black: Fmoc-YGFGL-NH_2 as reference. (Reaction time 120 min). Refer for legend of Fig. S2 for chromatographic conditions.

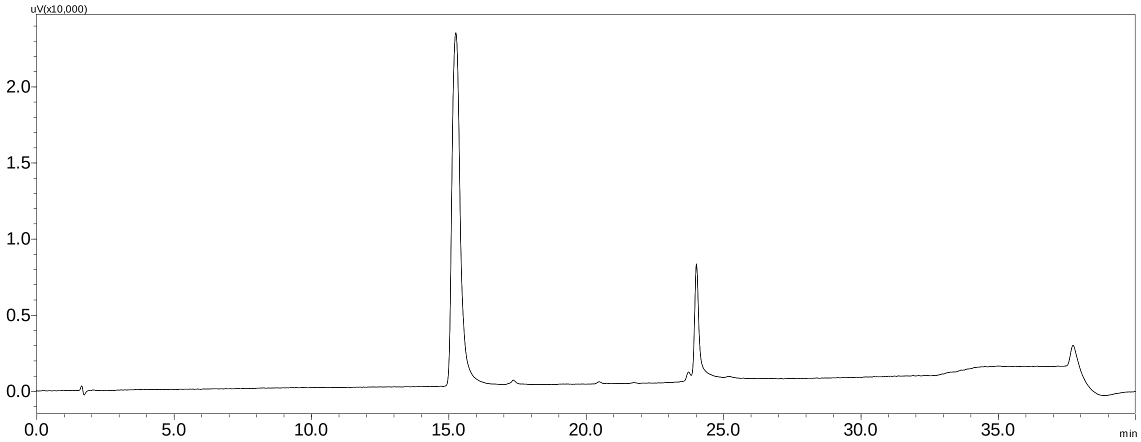

Supplementary Figure 28. Blank injection of anisole. Refer for legend of Fig. S2 for chromatographic conditions.

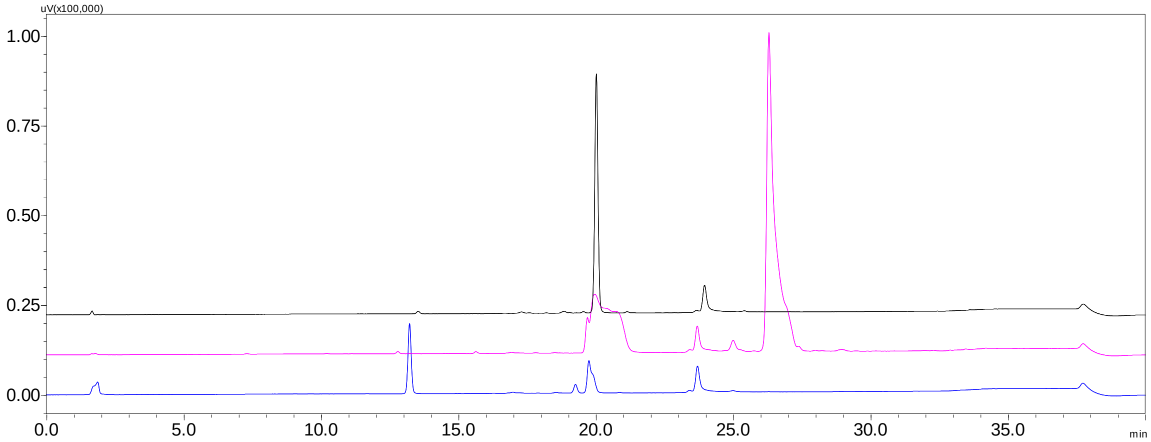

Supplementary Figure 29. Cleaved Fmoc-Y(tBu)GFGL-NH2. Blue: 2%TFA in toluene + 95% TFA; Pink: 2%TFA in toluene; Black: Fmoc-YGFGL-NH_2 as reference. (Reaction time 120 min). Refer for legend of Fig. S2 for chromatographic conditions.

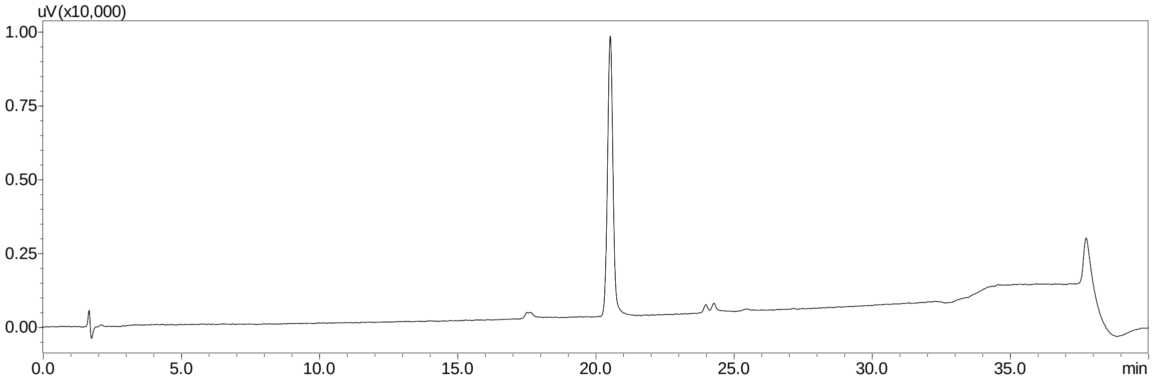

Supplementary Figure 30. Blank injection of toluene. Refer for legend of Fig. S2 for chromatographic conditions.

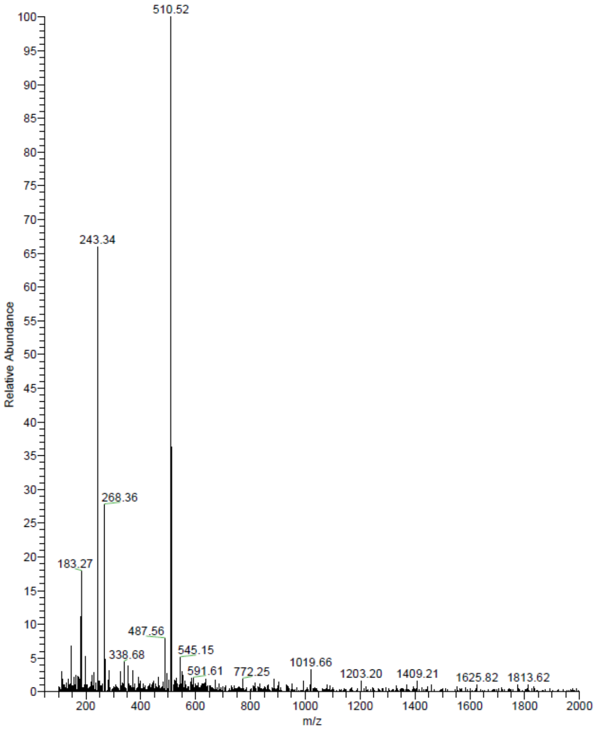

Supplementary Figure 31. Mass of H-H(Trt)L-NH_2. Calculated: 509.28, found: 510.52 $[M+H]^+$.

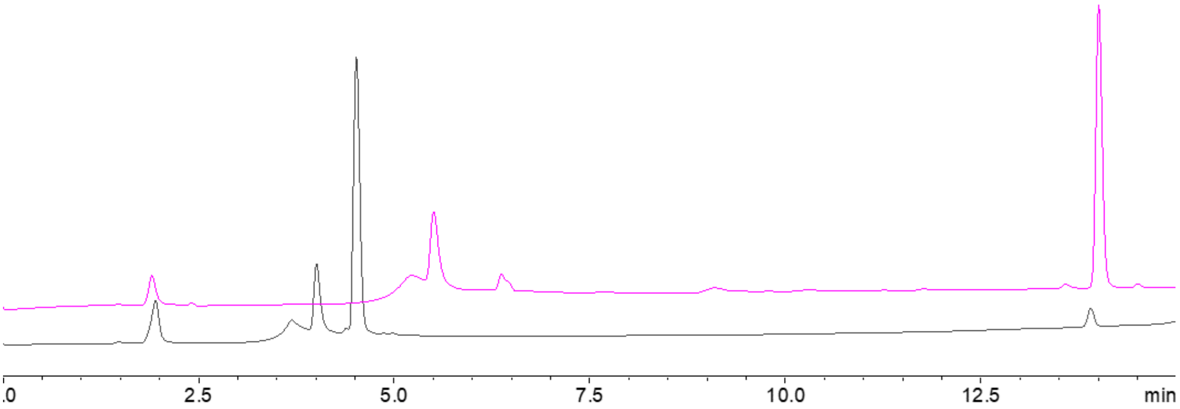

Supplementary Figure 32. Cleaved H-H(Trt)-L-NH_2. Pink: 2%TFA in DCM; Black: H-HL-NH_2 as reference. (Reaction time 120 min). λ = 220 nm. 0–50% in 15 min gradient elution. Refer for legend of Fig. S2 for chromatographic conditions.

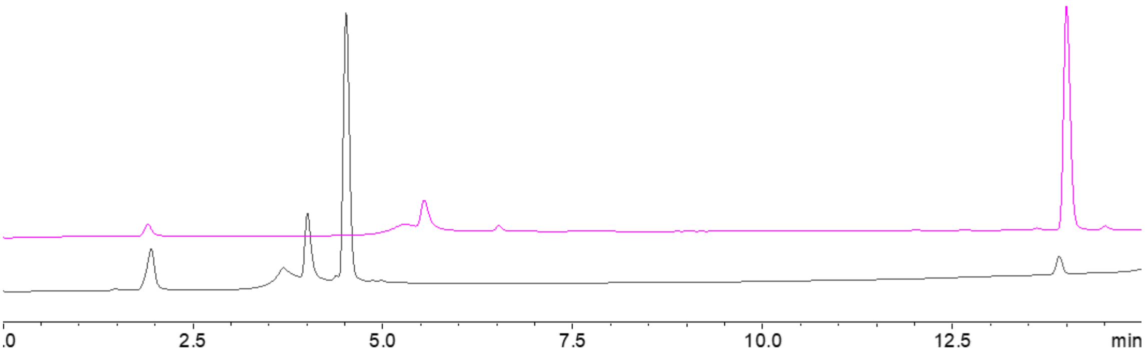

Supplementary Figure 33. Cleaved H-H(Trt)-LNH_2. Pink: 2%TFA in p-xylene; Black: H-HL-NH_2 as reference. (Reaction time 120 min). Refer for legend of Fig. S32 for chromatographic conditions.

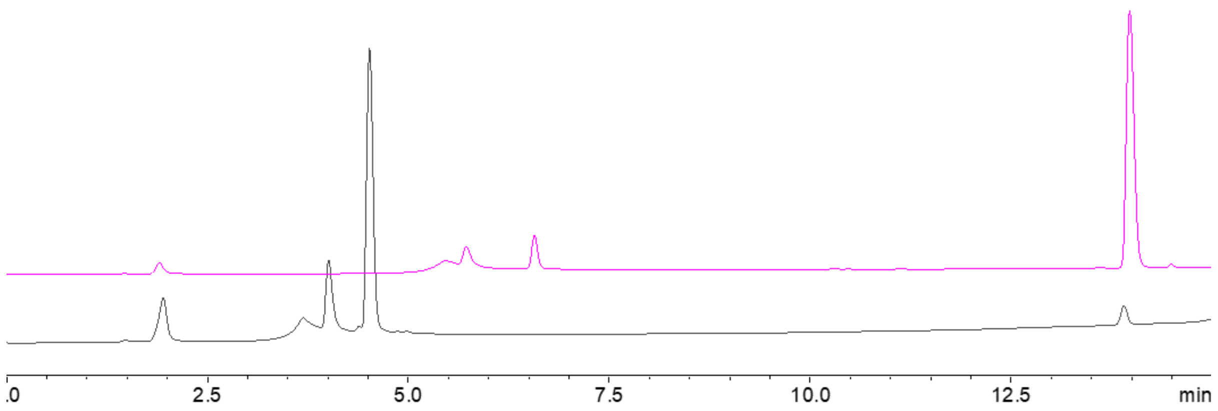

Supplementary Figure 34. Cleaved H-H(Trt)-L-NH_2. Pink: 2%TFA in toluene; Black: H-HL-NH_2. (Reaction time 120 min). Refer for legend of Fig. S32 for chromatographic conditions.

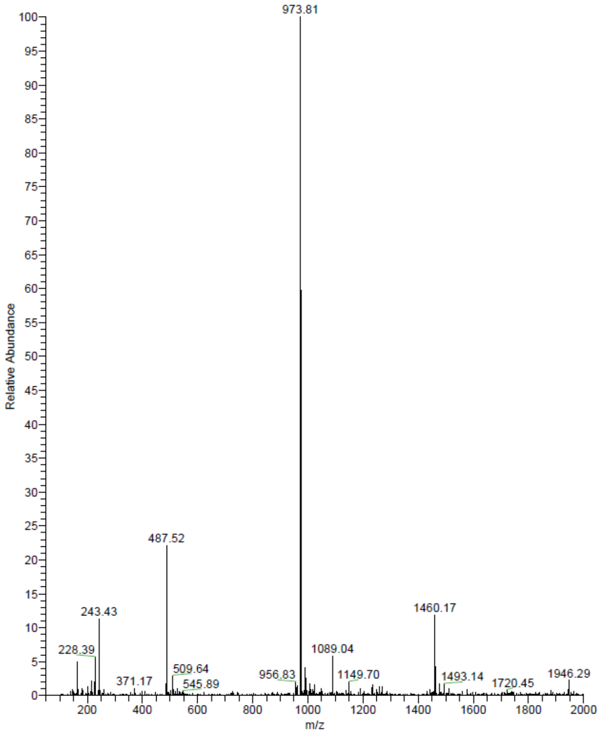

Supplementary Figure 35. Mass of H-N(Trt)L-NH_2. Calculated: 486.26 found: 487.52 $[M+H]^+$, 973.81 $[2M+H]^+$ (non-covalent dimer), 1460.17 $[3M+H]^+$ (non-covalent trimer).

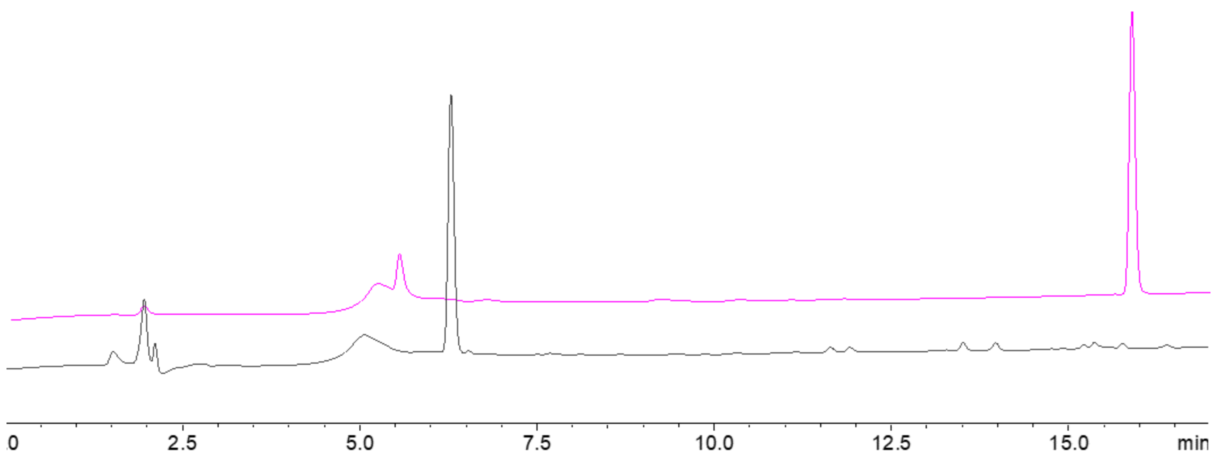

Supplementary Figure 36. Cleaved H-N(Trt)-L-NH_2. Pink: 2%TFA in DCM; Black: H-NL-NH_2 as reference. (Reaction time 120 min). Refer for legend of Fig. S32 for chromatographic conditions.

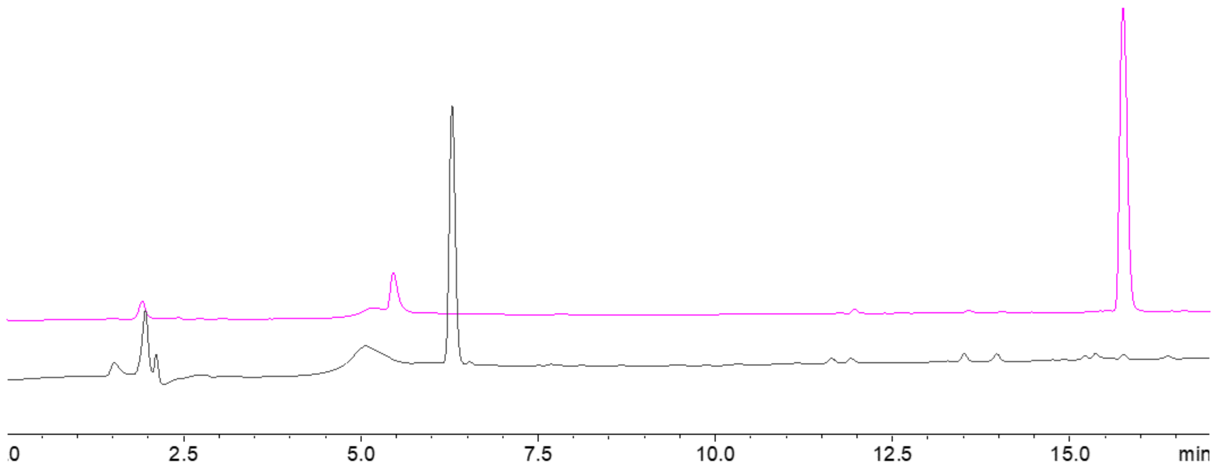

Supplementary Figure 37. Cleaved H-N(Trt)-L-NH_2. Pink: 2%TFA in p-xylene; Black: H-NL-NH_2 as reference. (Reaction time 120 min). Refer for legend of Fig. S32 for chromatographic conditions.

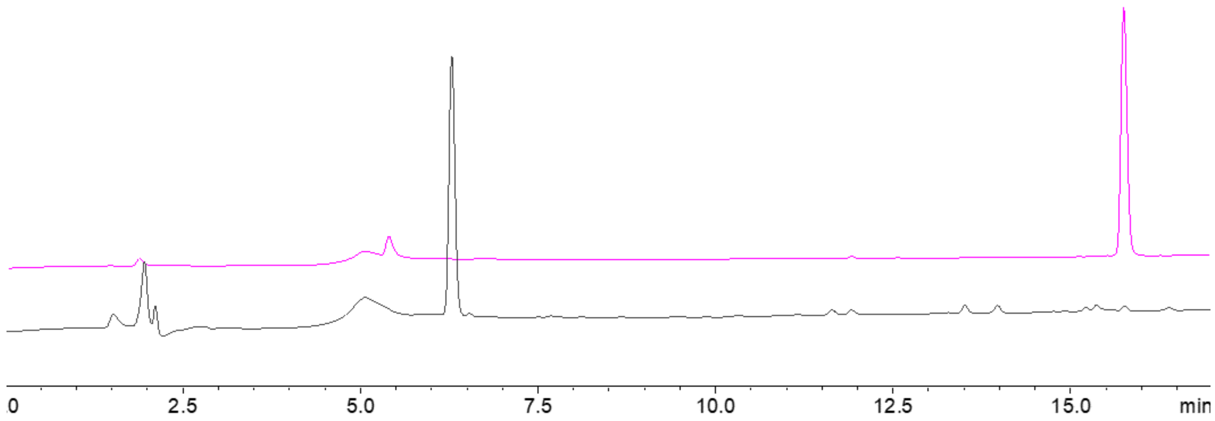

Supplementary Figure 38. Cleaved H-N(Trt)-L-NH_2. Pink: 2%TFA in toluene; Black: H-NL-NH_2. (Reaction time 120 min). Refer for legend of Fig. S32 for chromatographic conditions.

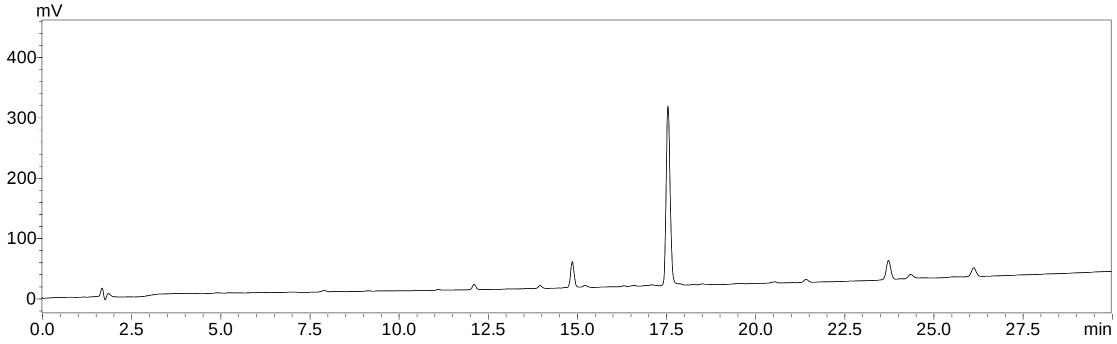

Supplementary Figure 39. Fmoc-Abaloparatide fragment. Refer for legend of Fig. S2 for chromatographic conditions.

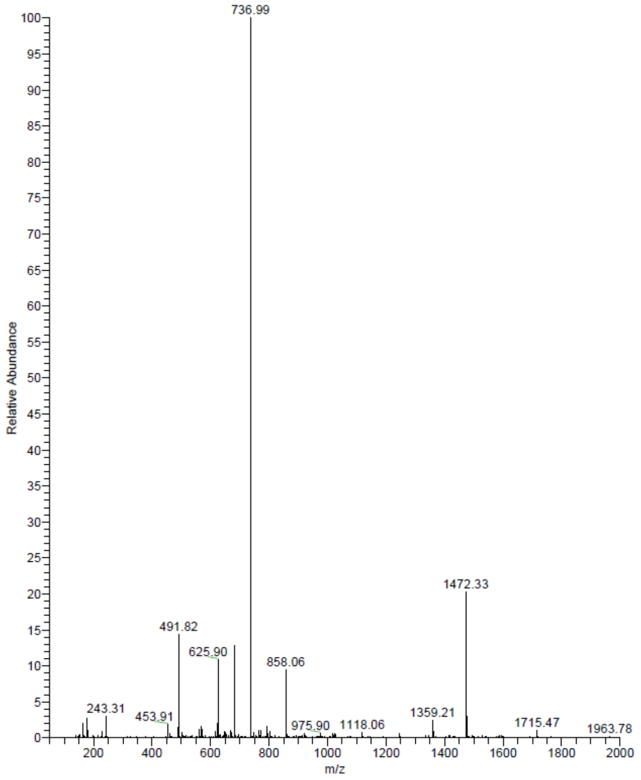

Supplementary Figure 40. Mass of Fmoc-Abaloparatide fragment. Calculated: 1471.81; found: 1472.33 $[M+H]^+$, 736.99 $[M+2H]^{2+}$.

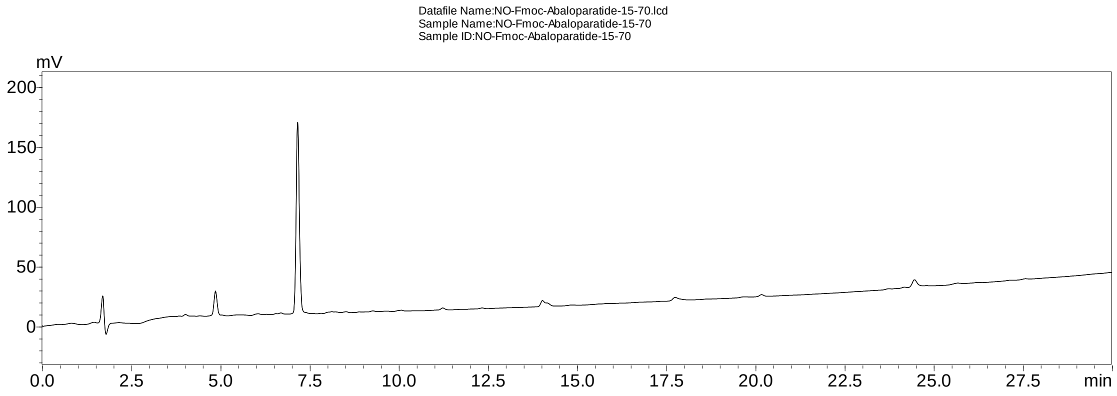

Supplementary Figure 41. Abaloparatide fragment. Refer for legend of Fig. S2 for chromatographic conditions.

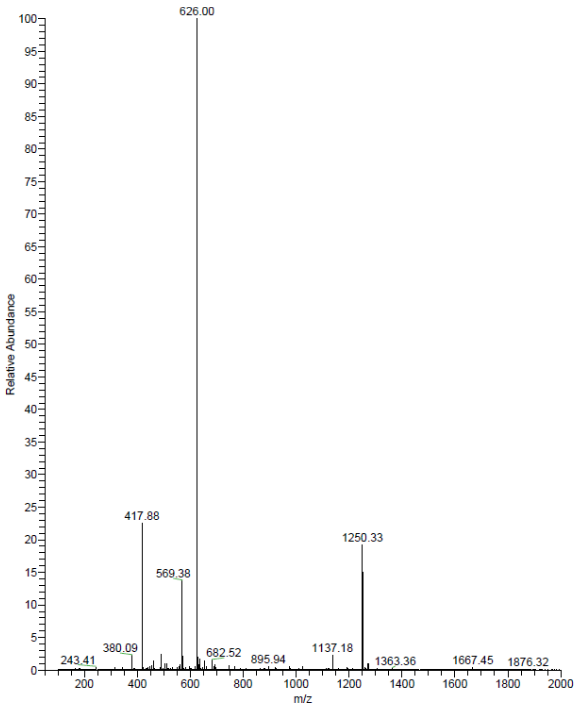

Supplementary Figure 42. Mass of Abaloparatide fragment. Calculated: 1249.57; found: 1250.33 $[M+H]^+$, 626.00 $[M+2H]^{2+}$, 417.88 $[M+3H]^{3+}$.

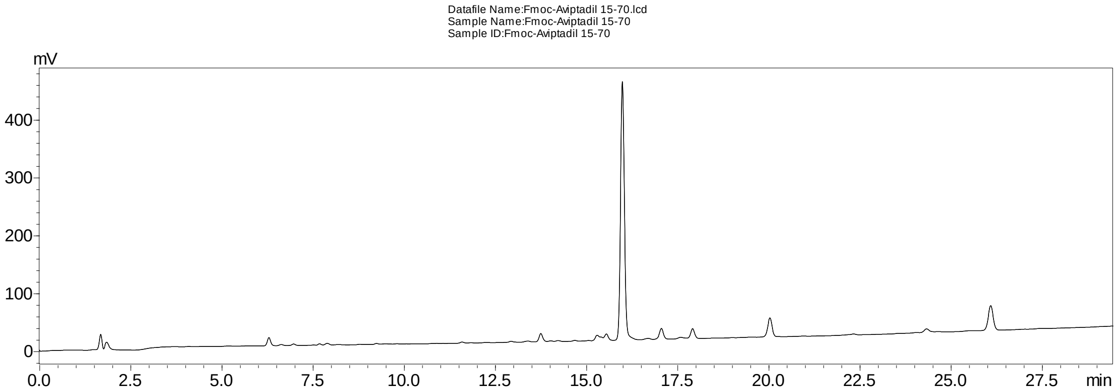

Supplementary Figure 43. Fmoc-Aviptadil fragment. Refer for legend of Fig. S2 for chromatographic conditions.

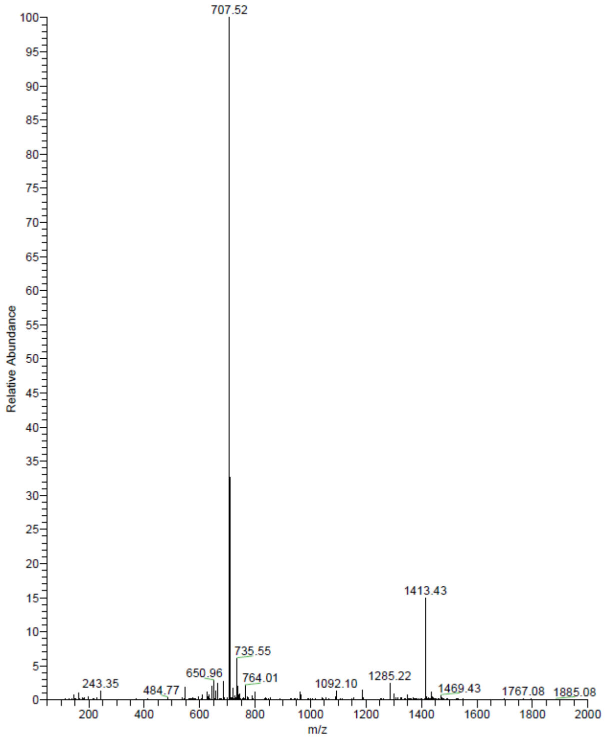

Supplementary Figure 44. Mass of Fmoc-Aviptadil fragment. Calculated: 1412.7; found: 1413.43 $[M+H]^+$, 707.52 $[M+2H]^{2+}$.

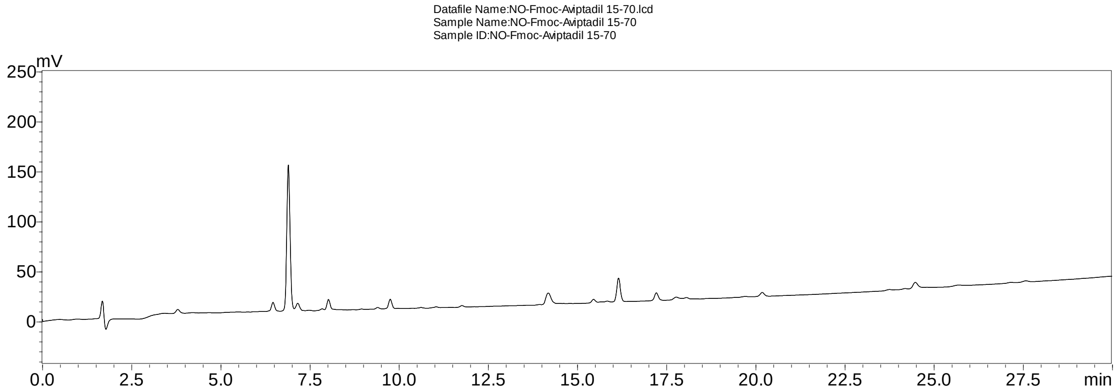

Supplementary Figure 45. Aviptadil fragment. Refer for legend of Fig. S2 for chromatographic conditions.

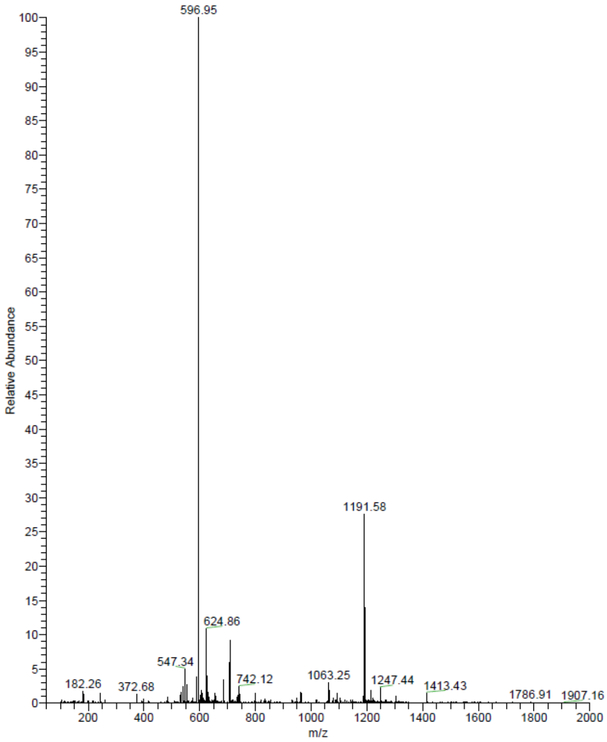

Supplementary Figure 46. Mass of Aviptadil fragment. Calculated: 1190.46; found: 1191.58 $[M+H]^+$, 596.95 $[M+2H]^{2+}$.

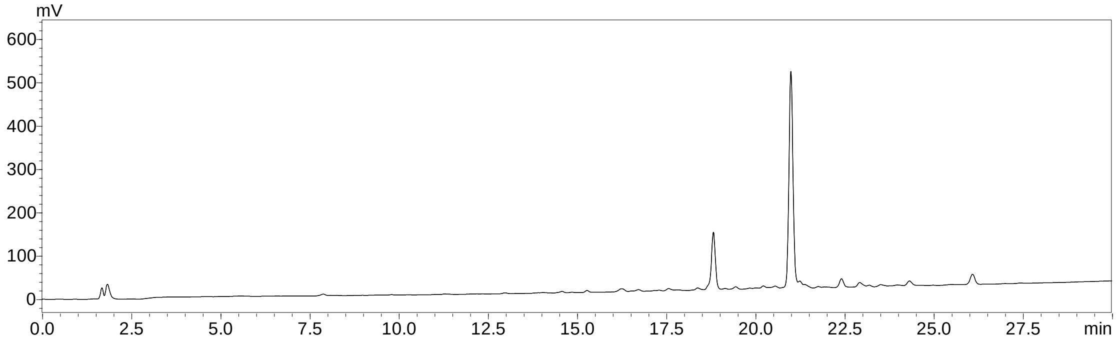

Supplementary Figure 47. Fmoc-T20 fragment. Refer for legend of Fig. S2 for chromatographic conditions.

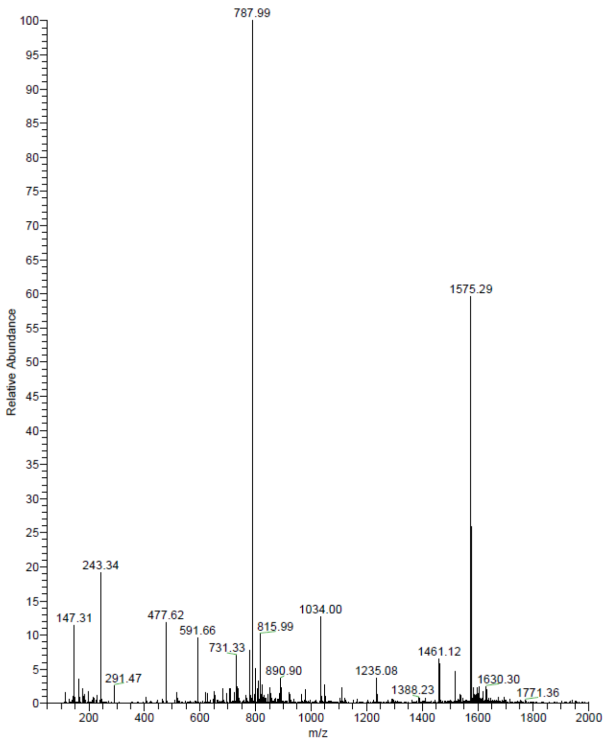

Supplementary Figure 48. Mass of Fmoc-T-20. Calculated: 1574.76; found: 1575.29 $[M+H]^{+}$, 787.99 $[M+2H]^{2+}$

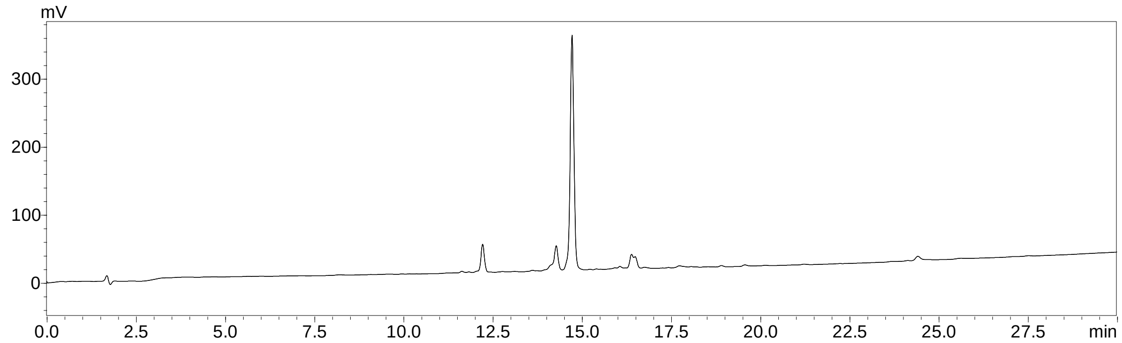

Supplementary Figure 49. T20 fragment. Refer for legend of Fig. S2 for chromatographic conditions.

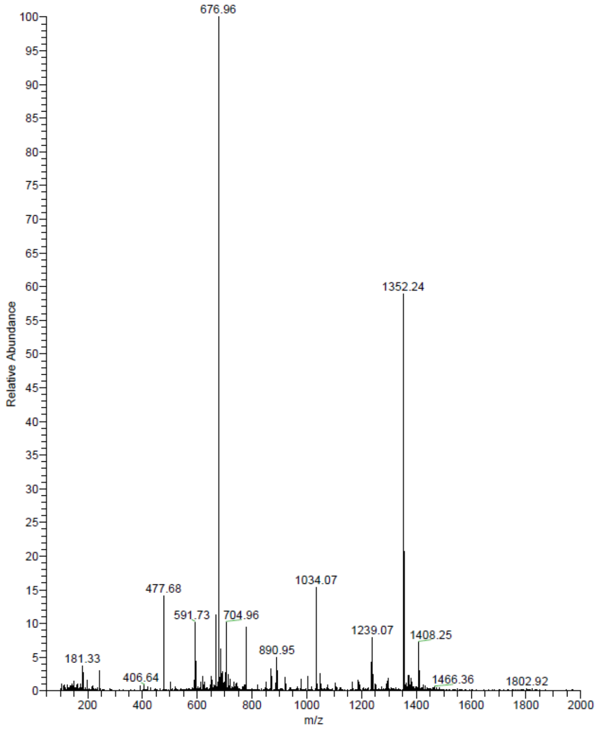

Supplementary Figure 50. Mass of T-20. Calculated: 1351.63; found: 1352.24 $[M+H]^+$, 676.96 $[M+2H]^{2+}$.

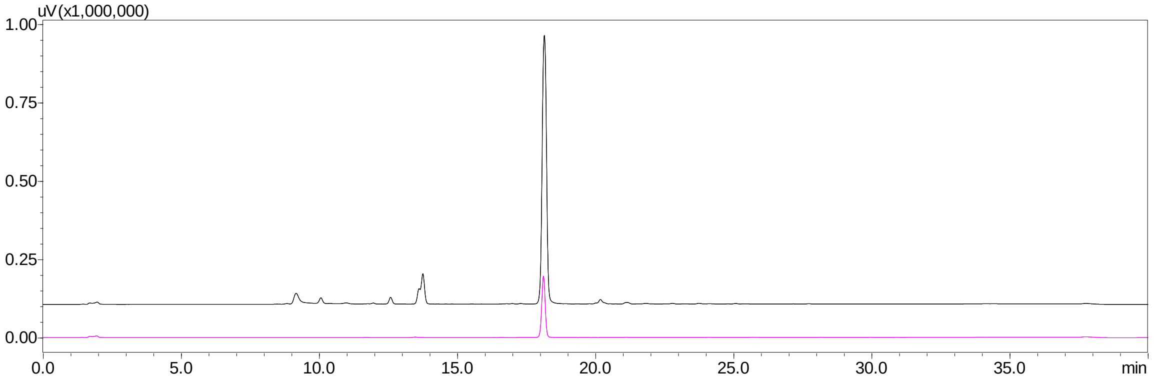

Supplementary Figure 51. Sieber amide native resin treated with TFA–TIS–H_2O (95:2.5:2.5). Black: Reaction time 120 min; Pink: Reaction time 60 min. Refer for legend of Fig. S2 for the chromatographic conditions.

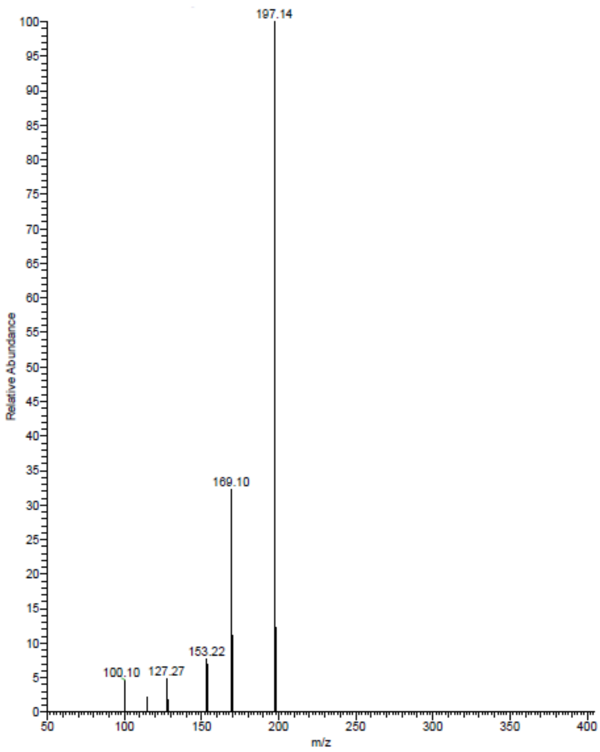

Supplementary Figure 52. Mass of Sieber amide linker: 215.25; found: 197.14 $[M-18]^+$.

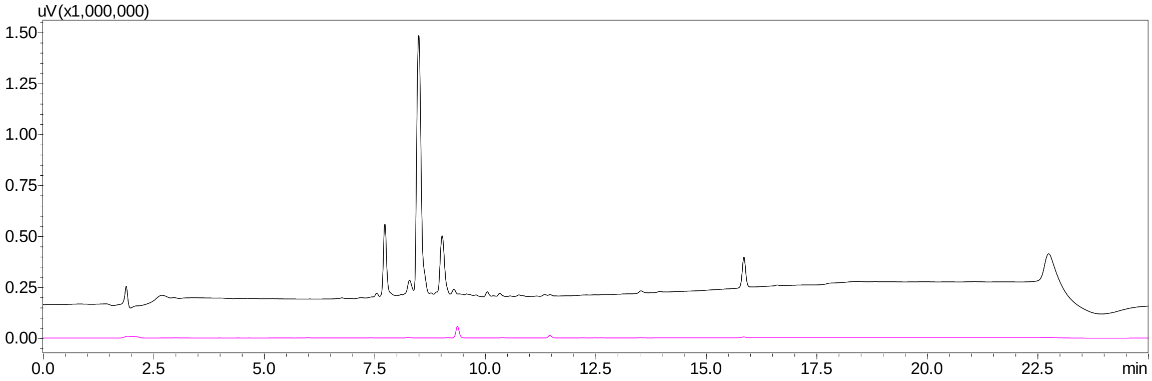

Supplementary Figure 53. Fmoc-Abaloparatide fragment. Black: Fmoc-LEKLLAibKLHTA-NH_2 as reference; Pink: 2% TFA in DCM + 95% TFA Fmoc- LE(*t*Bu)K(Boc)LLAibK(Boc)LH(Trt)T(*t*Bu)A-NH_2. Refer for legend of Fig. S2 for the chromatographic conditions.

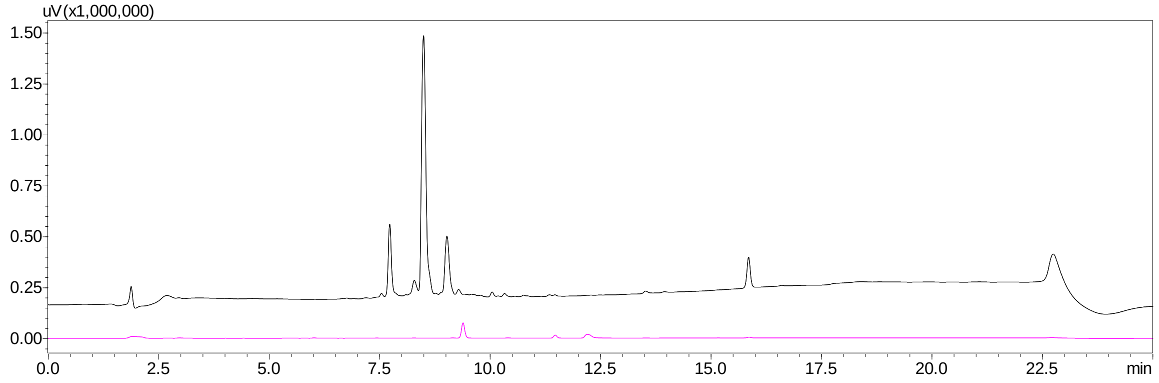

Supplementary Figure 54. Fmoc-Abaloparatide fragment. Black: Fmoc-LEKLLAibKLHTA-NH_2 as reference; Pink: 2% TFA in toluene + 95% TFA Fmoc- LE(*t*Bu)K(Boc)LLAibK(Boc)LH(Trt)T(*t*Bu)A-NH_2. Refer for legend of Fig. S2 for the chromatographic conditions.

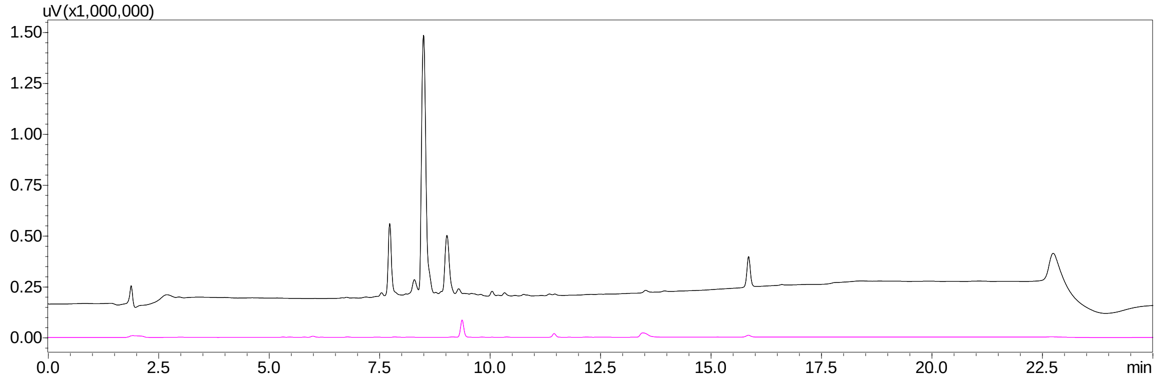

Supplementary Figure 55. Fmoc-Abaloparatide fragment. Black: Fmoc-LEKLLAibKLHTA-NH_2 as reference; Pink: 2% TFA in p-xylene + 95% TFA Fmoc- LE(*t*Bu)K(Boc)LLAibK(Boc)LH(Trt)T(*t*Bu)A-NH_2. Refer for legend of Fig. S2 for the chromatographic conditions.

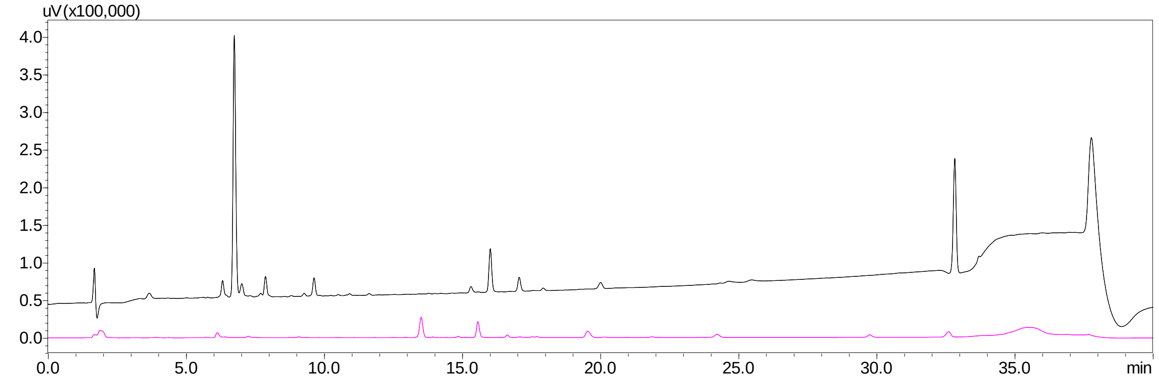

Supplementary Figure 56. Aviptadil fragment. Black: H-VKKYLNSILN-NH_2 as reference; Pink: 2% TFA in DCM + 95% TFA H- VK(Boc)K(Boc)Y(*t*Bu)LN(Trt)S(*t*Bu)ILN(Trt)-NH_2. Refer for legend of Fig. S2 for the chromatographic conditions.

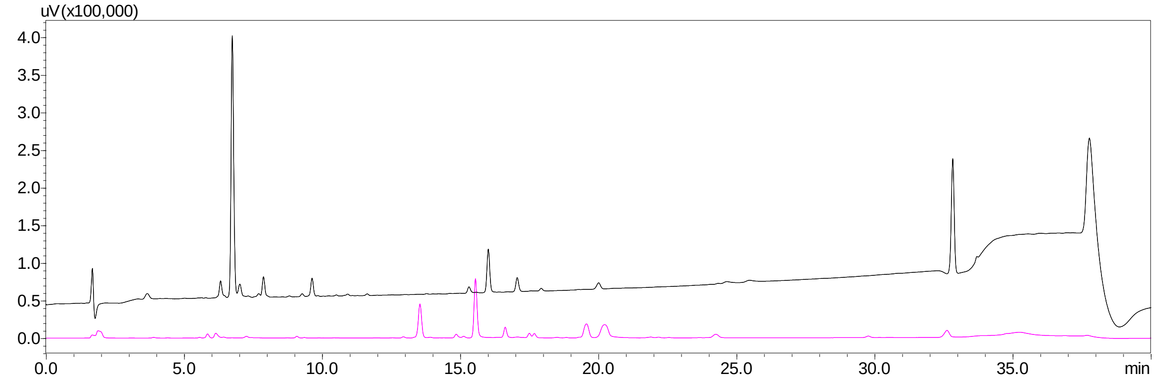

Supplementary Figure 57. Aviptadil fragment. Black: H-VKKYLNSILN-NH_2 as reference; Pink: 2% TFA in toluene + 95% TFA H- VK(Boc)K(Boc)Y(*t*Bu)LN(Trt)S(*t*Bu)ILN(Trt)-NH_2. Refer for legend of Fig. S2 for the chromatographic conditions.

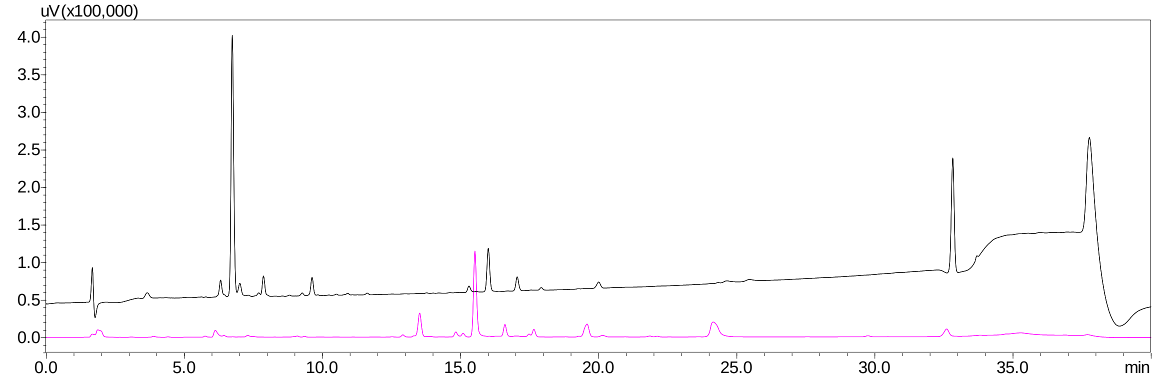

Supplementary Figure 58. Aviptadil fragment. Black: H-VKKYLNSILN-NH_2 as reference; Pink: 2% TFA in p-xylene + 95% TFA H- VK(Boc)K(Boc)Y(*t*Bu)LN(Trt)S(*t*Bu)ILN(Trt)-NH_2. Refer for legend of Fig. S2 for the chromatographic conditions.

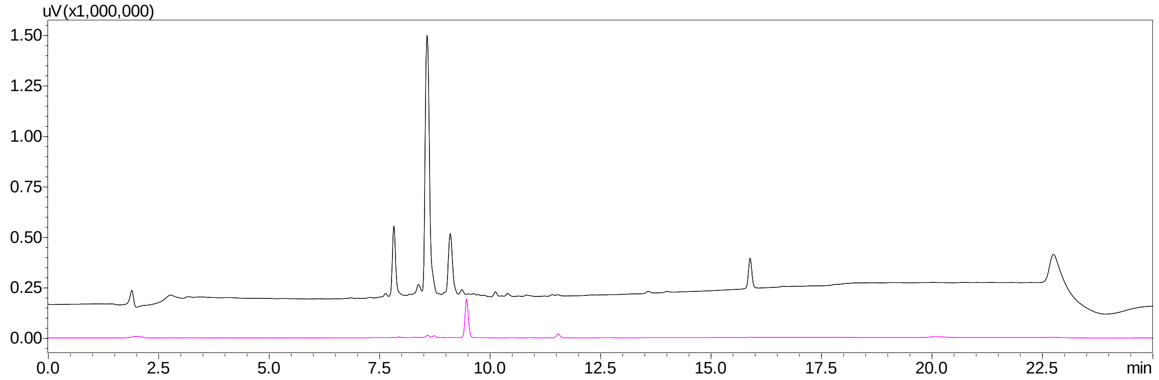

Supplementary Figure 59. T20 fragment. Black: H-DKWASLWNWF-NH_2 as reference; Pink: 2% TFA in DCM + 95% TFA H-D(*t*Bu)K(Boc)W(Boc)AS(*t*Bu)LW(Boc)N(Trt)W(Boc)F-NH_2. 5–95% in 15 min gradient elution. λ = 300 nm. Refer for legend of Fig. S2 for the rest of chromatographic conditions.

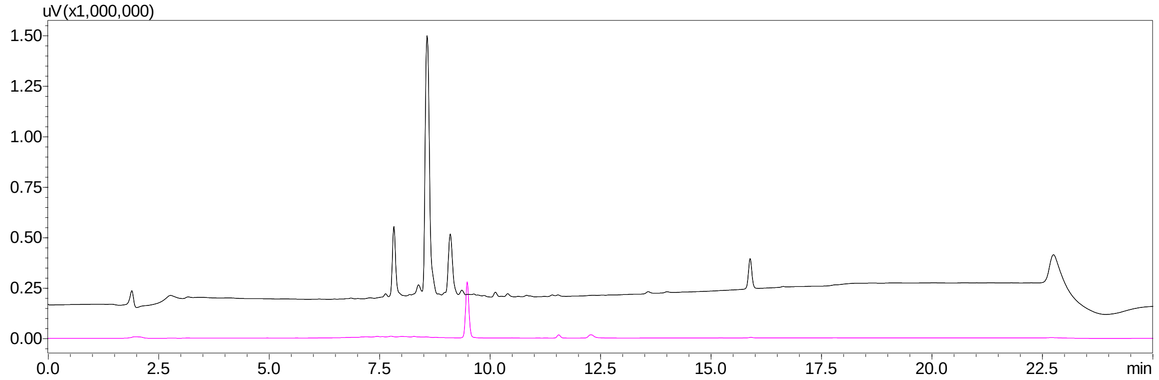

Supplementary Figure 60. T20 fragment. Black: H-DKWASLWNWF-NH_2 as reference; Pink: 2% TFA in toluene + 95% TFA H-D(*t*Bu)K(Boc)W(Boc)AS(*t*Bu)LW(Boc)N(Trt)W(Boc)F-NH_2. Refer for legend of Fig. S59 for the chromatographic conditions.

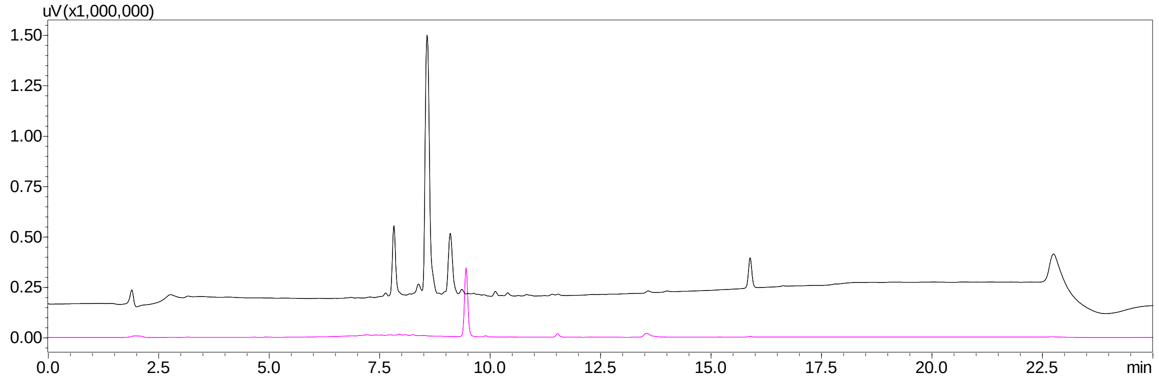

Supplementary Figure 61. T20 fragment. Black: H-DKWASLWNWF-NH_2 as reference; Pink: 2% TFA in p-xylene + 95% TFA H-D(*t*Bu)K(Boc)W(Boc)AS(*t*Bu)LW(Boc)N(Trt)W(Boc)F-NH_2. Refer for legend of Fig. S59 for the chromatographic conditions.

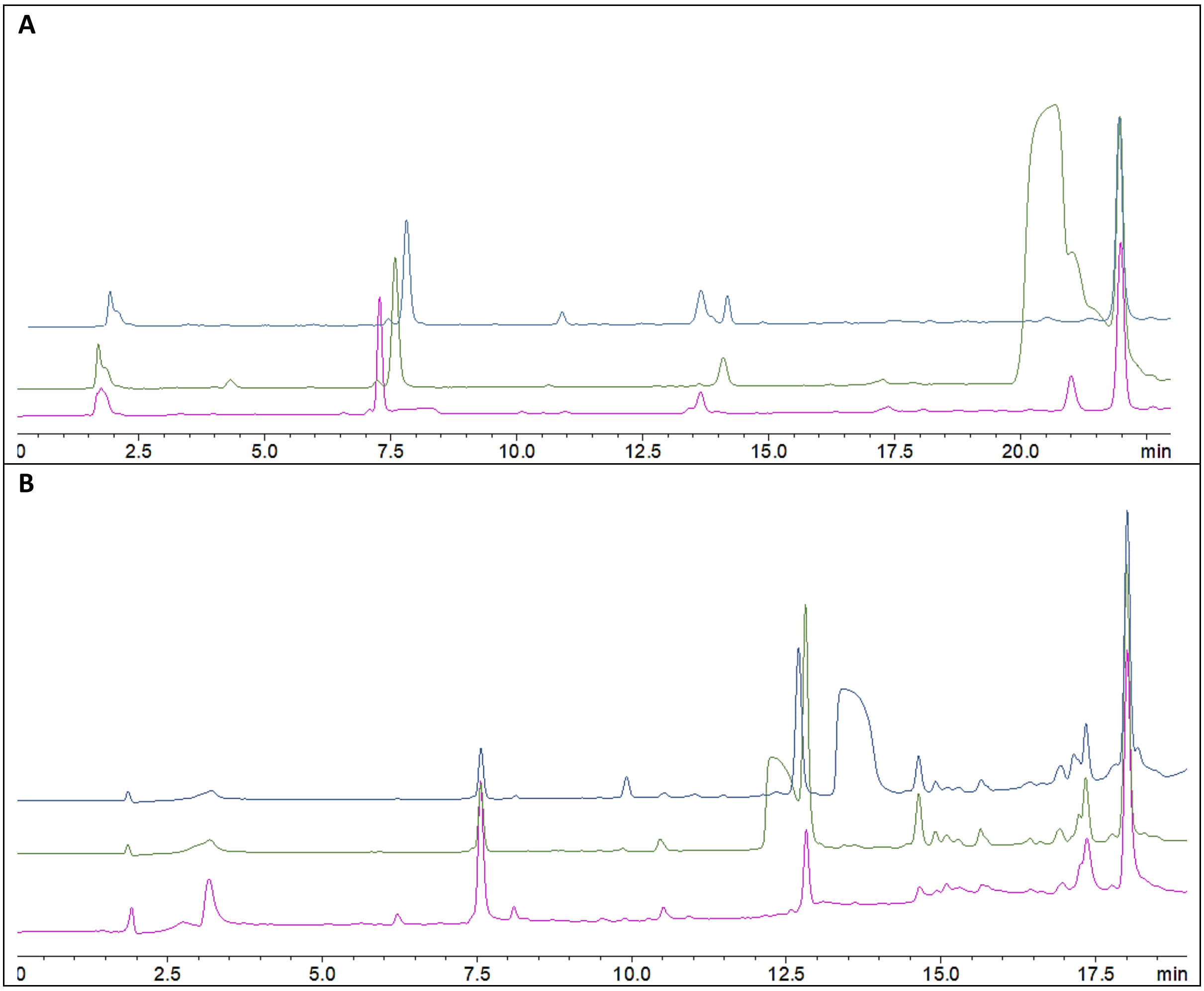

Supplementary Figure 62. Protected peptides: A. Fmoc-Aviptadil fragment: Fmoc-VK(Boc)K(Boc)Y(*t*Bu)LN(Trt)S(*t*Bu)ILN(Trt)-NH_2. B. Fmoc-T20 fragment: Fmoc-D(*t*Bu)K(Boc)W(Boc)AS(*t*Bu)LW(Boc)N(Trt)W(Boc)F-NH_2. Pink: 2%TFA in DCM; Green: 2%TFA in toluene; Blue: 2%TFA in p-xylene. (Reaction time 120 min). 15–70% in 30 min gradient elution for Fmoc-Aviptadil fragment and 5-95% in 15 min for Fmoc-T20. λ = 300 nm. Refer for legend of Fig. S2 for the rest of chromatographic conditions.

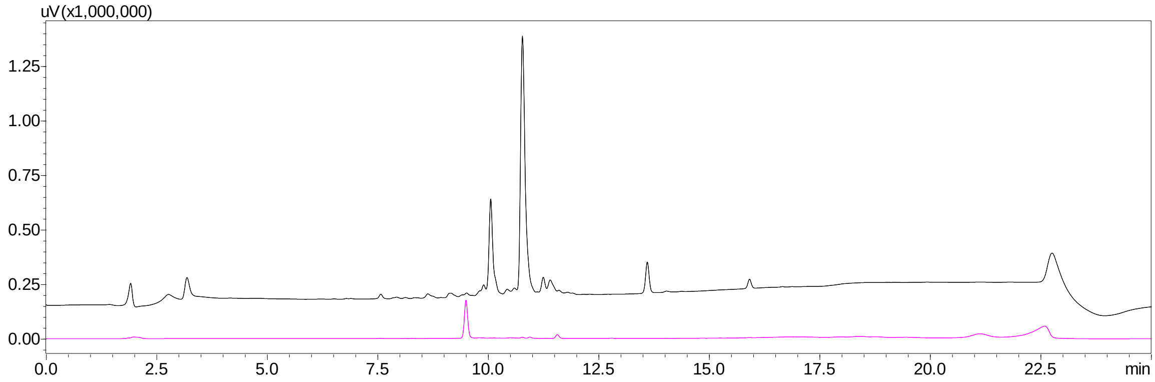

Supplementary Figure 63. Fmoc-T20 fragment. Black: Fmoc-DKWASLWNWF-NH_2 as reference; Pink: 2% TFA in DCM + 95% TFA Fmoc-D(*t*Bu)K(Boc)W(Boc)AS(*t*Bu)LW(Boc)N(Trt)W(Boc)F-NH_2. Refer for legend of Fig. S59 for the chromatographic conditions.

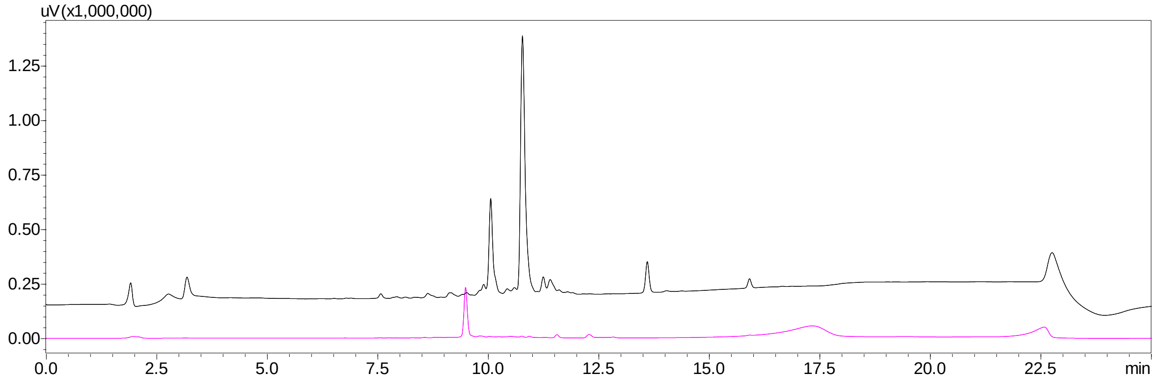

Supplementary Figure 64. Fmoc-T20 fragment. Black: Fmoc-DKWASLWNWF-NH_2 as reference; Pink: 2% TFA in toluene + 95% TFA Fmoc-D(*t*Bu)K(Boc)W(Boc)AS(*t*Bu)LW(Boc)N(Trt)W(Boc)F-NH_2. Refer for legend of Fig. S59 for the chromatographic conditions.

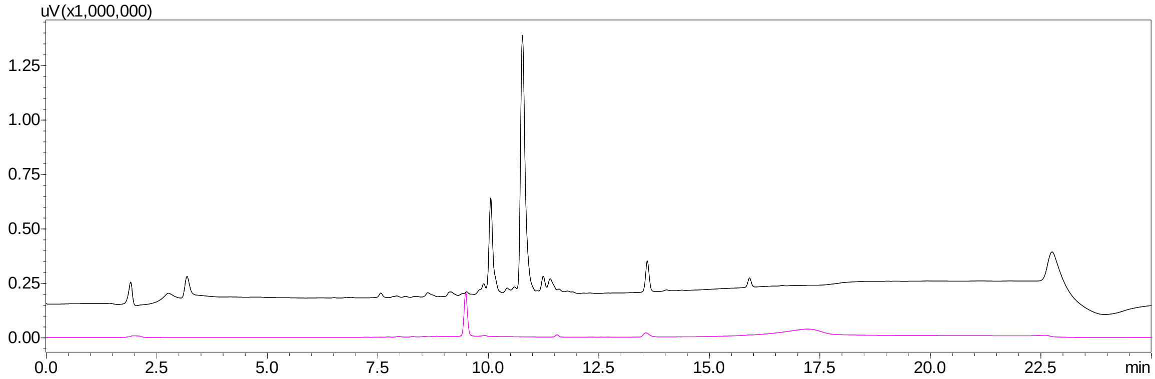

Supplementary Figure 65. Fmoc-T20 fragment. Black: Fmoc-DKWASLWNWF-NH_2 as reference; Pink: 2% TFA in p-xylene + 95% TFA Fmoc-D(*t*Bu)K(Boc)W(Boc)AS(*t*Bu)LW(Boc)N(Trt)W(Boc)F-NH_2. Refer for legend of Fig. S59 for the chromatographic conditions.

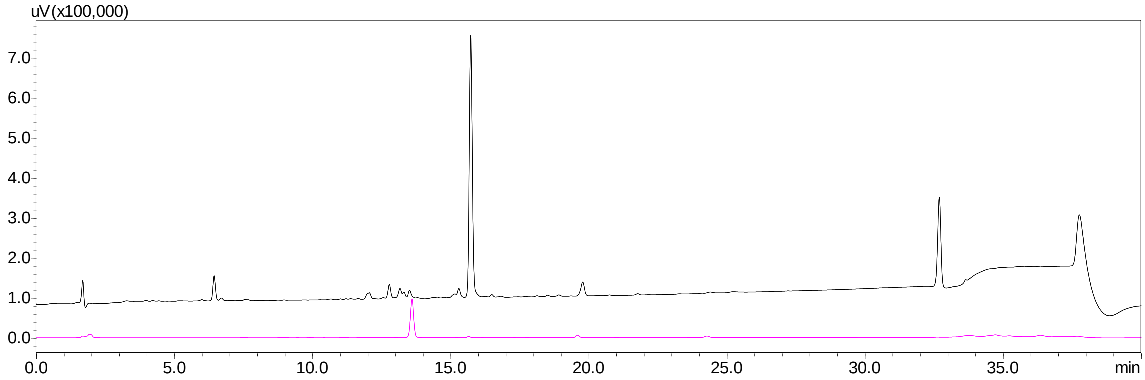

Supplementary Figure 66. Fmoc-Aviptadil fragment. Black: Fmoc-VKKYLNSILN-NH_2 as reference; Blue: 2%TFA in DCM + 95% TFA Fmoc- VK(Boc)K(Boc)Y(*t*Bu)LN(Trt)S(*t*Bu)ILN(Trt)-NH_2. Refer for legend of Fig. S2 for the chromatographic conditions.

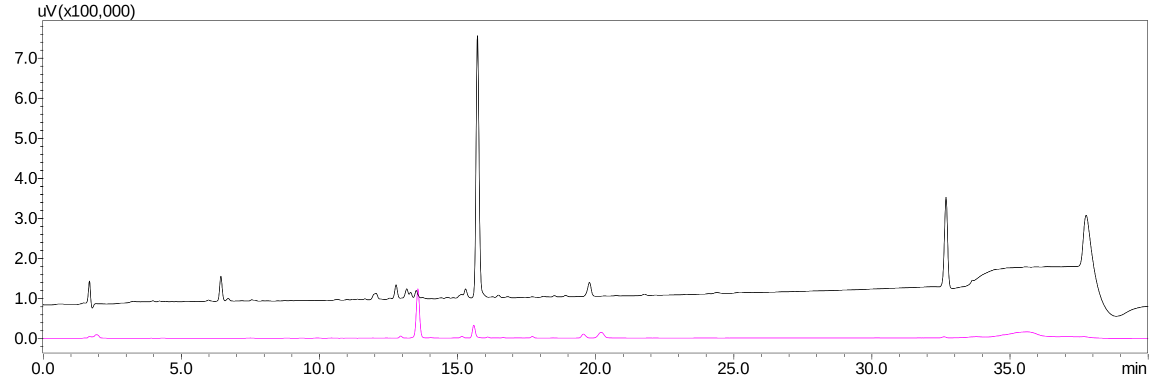

Supplementary Figure 67. Fmoc-Aviptadil fragment. Black: Fmoc-VKKYLNSILN-NH_2 as reference; Blue: 2%TFA in toluene + 95% TFA Fmoc- VK(Boc)K(Boc)Y(*t*Bu)LN(Trt)S(*t*Bu)ILN(Trt)-NH_2. Refer for legend of Fig. S2 for the chromatographic conditions.

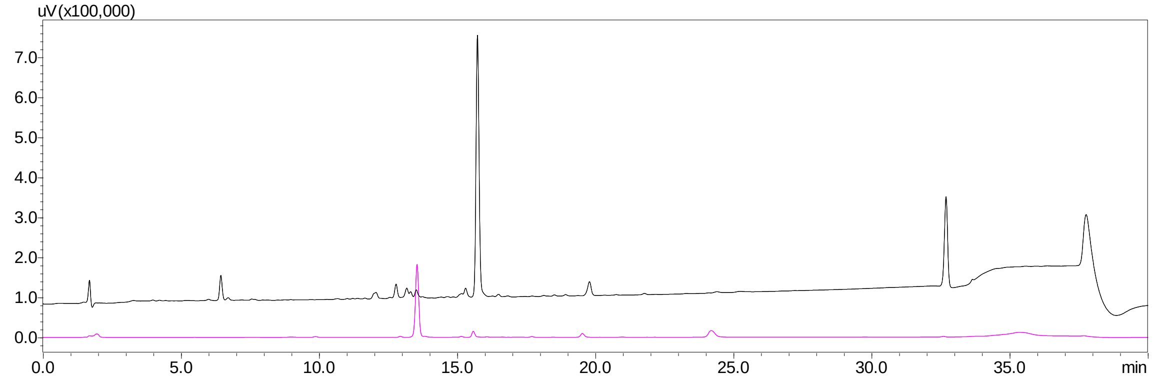

Supplementary Figure 68. Fmoc-Aviptadil fragment. Black: Fmoc-VKKYLNSILN-NH_2 as reference; Blue: 2%TFA in p-xylene + 95% TFA Fmoc- VK(Boc)K(Boc)Y(*t*Bu)LN(Trt)S(*t*Bu)ILN(Trt)-NH_2. Refer for legend of Fig. S2 for the chromatographic conditions.
